# Is CRISPR/Cas9 a way forward to fast-track genetic improvement in commercial palms? Prospects and limits

**DOI:** 10.3389/fpls.2022.1042828

**Published:** 2022-12-12

**Authors:** Faiza Shafique Khan, Farhan Goher, Dapeng Zhang, Peng Shi, Zhiying Li, Yin Min Htwe, Yong Wang

**Affiliations:** ^1^ Hainan Key Laboratory for Biosafety Monitoring and Molecular Breeding in Off-Season Reproduction Regions/Sanya Research Institute of Chinese Academy of Tropical Agricultural Sciences, Sanya, Hainan, China; ^2^ Hainan Yazhou Bay Seed Laboratory, Sanya, Hainan, China; ^3^ Hainan Key Laboratory of Tropical Oil Crops Biology, Coconut Research Institute of Chinese Academy of Tropical Agricultural Sciences, Wenchang, Hainan, China; ^4^ State Key Laboratory of Crop Stress Biology for Arid Areas, College of Plant Protection, Northwest A&F University, Yangling, Shaanxi, China

**Keywords:** coconut, date palm, genome editing, genetic improvement, oil palm

## Abstract

Commercially important palms (oil palm, coconut, and date palm) are widely grown perennial trees with tremendous commercial significance due to food, edible oil, and industrial applications. The mounting pressure on the human population further reinforces palms’ importance, as they are essential crops to meet vegetable oil needs around the globe. Various conventional breeding methods are used for the genetic improvement of palms. However, adopting new technologies is crucial to accelerate breeding and satisfy the expanding population’s demands. CRISPR/Cas9 is an efficient genome editing tool that can incorporate desired traits into the existing DNA of the plant without losing common traits. Recent progress in genome editing in oil palm, coconut and date palm are preliminarily introduced to potential readers. Furthermore, detailed information on available CRISPR-based genome editing and genetic transformation methods are summarized for researchers. We shed light on the possibilities of genome editing in palm crops, especially on the modification of fatty acid biosynthesis in oil palm. Moreover, the limitations in genome editing, including inadequate target gene screening due to genome complexities and low efficiency of genetic transformation, are also highlighted. The prospects of CRISPR/Cas9-based gene editing in commercial palms to improve sustainable production are also addressed in this review paper.

## 1 Introduction

The oil palm (*Elaeis guineensis* Jacq.), coconut (*Cocos nucifera* L.), and date palm (*Phoenix dactylifera* L.) are perennial, prominent Arecaceae (palm) family members. The Arecaceae family comprises over 2500 species in 200 genera and is ranked third after the Poaceae and Fabaceae (Meerow et al., 2012). The coconut, date palm, and oil palm trees have high commercial significance among palm trees (www.britannica.com/plant/palm-tree). Edible oil from an oil palm tree ranks first in the international vegetable oil market for its versatile usage in food, bioenergy, and industrial applications. Vegetable oil demand has risen dramatically during the last 50 years (Kumar et al., 2016). The world population is expanding abruptly and will reach up to 10.9 billion by the end of the 21^st^ century (Adam, 2021). The quantity of land dedicated to cultivating oil palm has risen substantially during the previous half-century (Ritchie and Roser, 2021). The global market is dominated by Indonesia and Malaysia, and accounts for 84% of edible oil production (FAO, 2018; Ritchie and Roser, 2020). Coconut has diverse uses, including oil, shell charcoal, husk fiber, and cortex production (Sayer et al., 2012; Perera, 2014). Coconut palm “Tree of Life” is grown in 90 countries worldwide, mostly in tropical areas (Adkins et al., 2006; FAO, 2018). Date palms are grown in arid conditions in the desert oasis. The primary date palm producing countries are Egypt, Iran, Saudi Arabia, UAE, Pakistan, Algeria, and Iraq, contributing up to 89% of date palm production (Manickavasagan et al., 2012; Aljohi et al., 2016; FAO, 2018; Salomón-Torres et al., 2021). Oil extraction from date palm seeds is a promising new source of edible oil for human consumption (Ali et al., 2015). However, considering their heterozygosity, perennial nature, and genome complexity, palm trees have been an ignored group for genome editing and genetic improvement compared to commercial plant species.

Numerous biotechnological tools with slight advancements in genetic transformation and *in vitro* propagation are used for genetic improvement in palm trees. The traditional breeding approaches are not cost-effective, as better performance in this system depends upon extensive backcrossing (Sattar et al., 2017; Sattar et al., 2021). The coconut palm has a long juvenile phase (5 – 9 years), and the lack of efficient vegetative propagation methods is the major constraint in breeding (Rajesh et al., 2021). Moreover, coconut palm propagation through seeds causes phenotypic variations within the plantation and hinders agronomic practices. Similarly, date palm backcrosses take 30 years (Sattar et al., 2017), and oil palm backcrosses 15–18 years (Babu et al., 2021). Keeping these above-mentioned scenarios in mind, adopting new technology is mandatory to develop a high-yielding, stress tolerant, and good fruit quality cultivar (Abdullah et al., 2017; Haque et al., 2018; Khan et al., 2022).

The artificial methods used for gene modification, overexpression, insertion, and deletion in the plant genetic material make it genetically modified (GM) (Bao et al., 2020; Shafique Khan et al., 2021). In present era, Clustered, regularly interspaced short palindromic repeat/CRISPR-associated protein 9 (CRISPR/Cas9) based gene editing (GE) and genetically modified organisms (GMOs) are the most promising tools in plant biotechnology. Genetically modified organisms contain foreign DNA, often from different species, that induces new traits (Sprink et al., 2016). On the other hand, CRISPR-based GE allows precise target changes in an organism’s existing DNA. The CRISPR/Cas9 technology has emerged as a breakthrough in genome editing that took off in 2013. Scientists are now using CRISPR-mediated GE to develop crops that allow farmers to produce more food sustainably (Li et al., 2013; Nekrasov et al., 2013; Shan et al., 2013). A successful CRISPR initiation in a model species (i.e. *Arabidopsis*) makes it a prominent technology for developing non-genetically modified (non-GM) crops with the desired traits. Genome editing through the CRISPR/Cas9 system is fast, accurate, and enables highly effective targeted GE. The CRISPR/Cas9 technique has been successfully utilized in various crops (Fister et al., 2018). Until now, CRISPR technology revealed an unprecedented impact on commercial agriculture production.

Due to their complex genomes, limitations in the genetic transformation system, and plant regeneration process, genome editing and genetic improvement in commercially significant palm trees have been difficult (Bekheet and Hanafy, 2011). Commercial applications of genetically modified trees or palms are so far limited. There is no report of genome editing in palm trees (coconut, date palm) (Budiani et al., 2018). Biotechnological techniques in palm tree genomics, such as marker-assisted breeding, DNA fingerprinting, and transgenics, are failed to improve the situation (Rohde et al., 2002; Singh et al., 2018). Mainly, CRISPR/Cas9-mediated genome editing in trees focuses on editing *Phytoene Desaturase* (*PDS*), which induces an albino phenotype due to reduced photosynthesis and the carotenoid pathway (**Table 1**) (Jia and Wang, 2014; Nishitani et al., 2016; Jia et al., 2017; Kaur et al., 2018). This evidence suggests that CRISPR/Cas9 could become a powerful tool for efficient genome editing (Bahariah et al., 2021). Despite the emerging prospects demonstrated in the below-mentioned studies (**Table 1**), coconut, oil palm, and date palm are still complex commercial trees with numerous challenges. These obstacles must be overcome before starting a large-scale genetic improvement program. In the present review, we focus on the primary options available for GE technologies, the application of proposed methods for genetic transformation, and regulatory pathways for genetic improvement in the palm family. We also highlight the bioengineering role of fatty acid biosynthesis in enhancing the commercial production of vegetable oil for human consumption. Finally, the subsequent prospects and limits of genome editing, and applications of CRISPR in the palms (date palm, coconut palm, and oil palm), have been briefly discussed in this review.

**Table 1:** CRISPR-based GE in trees.

Species	Target	Transformation method	Explant	CRISPR/Cas9 system	Promoter	Mutation frequency	Regeneration	Reference
Oil palm	*EgPDS*	Electroporation	Protoplast	CRISPR/Cas9	OsU3,UBI	62.5-83.33%.	Yes	(Yeap et al., 2021)
Oil palm	*EgBRI1*	Electroporation	Protoplast	CRISPR/Cas9	OsU3,UBI	58.82-100%.	Yes	(Yeap et al., 2021)
Grape	*VvPDS*	Agrobacterium	Embryonic calli	CRISPR/Cas9	AtU6-26,PcUbi4-2	7-72.2%	Yes	(Nakajima et al., 2017)
Banana	*RAS-PDS*	Agrobacterium	embryogenic cell	Single target	OsU3, 2×CaMV35S	59%	Yes	(Kaur et al., 2018)
Apple	*MdPDS*	Agrobacterium	Leaf disc	Single target	CaMV35S AtU6-1	13.6%	Yes	(Nishitani et al., 2016)
Sweet orange	*CsPDS*	Xcc-facilitated agroinfiltration	Leaves	Single target	CaMV35S	3.2-3.9%	No	(Jia and Wang, 2014)
Carrizo citrange	*CcPDS*	Agrobacterium	Epicotyls	Single target	AtYAO, AtU6-26	45.4-75%	Yes	(Zhang et al., 2017b)
Poplar	*PdPDS*	Agrobacterium	Leaf disc	Cas12a genome-targeting	AtU6-26, CaMV35S	70%	Yes	(An et al., 2020)
Kiwifruit	*AcPDS*	Agrobacterium	Leaf disc	PTG/Cas9	CaMV35S, AtU6-1	65.38-91.67%	Yes	(Wang et al., 2018)
Grape	*MLO-7*	PEG-mediated	Protoplast	DNA-free, (RNPs)	CaMV35S, AtU6	0.1%	No	(Malnoy et al., 2016)
Grape	*VvPDS*	Agrobacterium	Embryogenic cells	multiplex editing	AtU6, VvU6, VvU3	34.8-43.24	Yes	(Ren et al., 2021)
Grape	*VvTMT*	Agrobacterium	Embryogenic cells	PTG/Cas9	UBQ2, VvU6, U3	–	Yes	(Ren et al., 2021)
Grapefruit	*CsLOB1*	Xcc-facilitated agroinfiltration	Epicotyl	Single target	CaMV35S	23.80-89.36%	Yes	(Jia et al., 2017)
Apple	*DIPM*	PEG-mediated	Protoplast	DNA free (RNPs)	AtU6-1, CaMV35	0.5-6.9%	No	(Malnoy et al., 2016)

PDS, Phytoene desaturase; BRI1, Brassinosteroid-insensitive 1; MLO-7, mildew resistance locus o; TMT, Sugar-related tonoplastic monosaccharide transporter; DIPM, DspA/E-interacting proteins from Malus; LOB1, LATERAL ORGAN BOUNDARIES.

## 2 Recent developments in genome editing in palm trees

### 2.1 Oil palm

Significant efforts have been made to improve the oil palm’s commercial traits in response to market demands using conventional breeding and genetic engineering. Conventional breeding requires 15–18 years to release commercial oil palm varieties (Wahid et al., 2005; Weckx et al., 2019; Yue et al., 2021). Ploidy is a valuable tool for genetic variability and improvement in various fruiting trees (Sattler et al., 2016). Colchicine and oryzalin have been shown to increase the ploidy level in oil palm when applied to the seeds (Madon et al., 2005). Morphologically, tetraploid plantlets showed thick and dark green leaves and flowers compared to diploid (control) plantlets (Te-chato, 2012). Although ploidy is additive to oil palm and preliminary screened through morphological and physiological parameters. Flow cytometry is a quick and easy method for screening ploidy levels in oil palm trees (Te-chato, 2012). On the other hand, polyploidy often results in reduced fertility due to meiotic errors, allowing for the production of seedless varieties (Comai, 2005).

The Malaysian Palm Oil Board (MPOB) is a pioneer in developing genetically modified palm trees with high oleate content (Sambanthamurthi et al., 2002). In 2018, a Malaysian group attempted to use CRISPR/Cas9-mediated genetic improvement in oil palm resistance to *Ganoderma*. The CRISPR construct targeted the *methallothionine-like* and *isoflavone reductase* (*IFR*) genes in callus. (Budiani et al., 2018). Such an approach might provide new prospects for studies of crucial genes involved in disease resistance in palms. Recently, a group designed an expression cassette to target four genes. However, it is not well known whether the expression cassette and CRISPR/Cas9 vector successfully transformed into oil palm (Aprilyanto et al., 2019). Above mentioned studies report that CRISPR/Cas9 successfully use in oil palm genome editing (Yeap et al., 2021). In another study, the oil palm mesophyll protoplasts were extracted from unopened spear leaves (Masani et al., 2013). Protoplasts were co-transformed with vector constructs using the electroporation technique. The *EgPDS* gene is used as a phenotypic marker to enable the screening of mutant lines, as mutants of the *PDS* gene show an albino phenotype (**Table 1**). Moreover, the *brassinosteroid-insensitive 1* (*EgBRI1*) gene is used to assess mutagenesis efficacy and specificity resulting in nucleotide substitutions with an early necrosis phenotype. DNA insertions and deletions (InDels) in the *EgBRI1* gene cause a decreased plant elongation phenotype. At the same time, leaf apex necrosis is developed from base substitution events in transgenic palms (Yeap et al., 2021). In a recent study, the Nuzhdin Lab (USA), in partnership with the MPOB perform sequencing of more then 200 *E. oleifera* and *E. guineensis* palm trees from Southeast Asia, Africa, Central and South America (Gene bank accession PRJNA434010) (Ithnin et al., 2021). The above-mentioned reports, there are still some limitations in the prevailing protocols that need further improvement for trait-specific GE and regeneration of mutant plants. However, the advances in sequencing technologies have enabled the accessibility of whole-genome sequencing in palms.

### 2.2 Coconut

**Figure 1:**
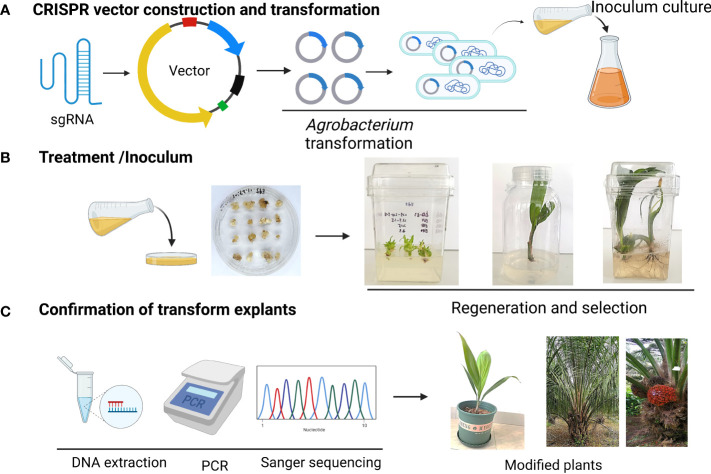
Schematic representation of CRISPR/Cas9 vector delivery tissue culture and selection strategy for genome-edited palm. **(A)** CRISPR vector construction and transformation **(B)** Treatment and infection to explants **(C)** Confirmation of positive plants.

Coconut is a diploid (2*n* = 32) cross-pollinating, valuable commercial tropical tree (Perera, 2014). To date, coconut breeding approaches for trait improvement have relied on conventional breeding methods (Lineesha and Antony, 2021). Progress in molecular biology and genomics has paved the way for gene identification and characterization. A recent publication by a group at the Coconut Research Institute, Chinese Academy of Tropical Agricultural Sciences (CATAS), provides a draft genome of coconut and basic genomic information to ease future functional genomics in coconut (Xiao et al., 2017). The availability of the coconut genome sequence (Lantican et al., 2019) gives us a valuable genetic resource to utilize GE tools to achieve precise DNA modification (**Figure 1**). To date, genome editing has rarely been studied in coconut. However, the developments in the field of genome editing offer optimism for revealing the potential of the coconut genome.

### 2.3 Date palm

The date palm is a diploid (2*n* = 36) dioecious tree with a significant economic value. However, breeding progress in date palm is slow due to various challenges. The availability of a complete genome opened an opportunity for genetic improvement in date palm. The genetic map for the date palm cultivar ‘Khalas’ was constructed a few years back (Mathew et al., 2015). The date palm genome was updated by researchers at NYU Abu Dhabi’s Center for Genomics and Systems Biology (NYUAD-CGSB) and the UAE Khalifa Center for Genetic Engineering and Biotechnology (KCGEB) (Yang et al., 2010; Hazzouri et al., 2019). Moreover, in the so-called “multi-omics analysis”, data on valuable traits is accumulating quickly in other commercial trees (Khan et al., 2022), as well as in date palms (Subhi et al., 2017). Whole-genome sequence data is required for genome editing and breeding systems to commercialize palm trees with newly acquired traits. (Yarra et al., 2020). The *DnMRE11* gene triggers DSB repair in the date palm cultivar ‘Deglet Noor’. Gene*DnMRE11* can be used as a dynamic tool for CRISPR/Cas9-based genome editing (Stracker and Petrini, 2011; Rekik et al., 2015). Marker-assisted technique improves the genetic identity and sex determination in seedlings. Markers such as mPdIRD52 and DPM4 are reported for sex determination in date palm. These markers showed 100% accuracy in sex determination in male and female plants at the nursery stage (Wang et al., 2020). Moreover, the gene-knocking ability of CRISPR/Cas9 technology can evaluate sex-determination genes in date palms (Sattar et al., 2017). Many agronomically significant loci are located in the “SNP deserts” (Xu et al., 2012; Hazzouri et al., 2015). Compared to the whole genome, the SNP dessert in the date palm genome recruits a high density of abiotic and biotic resistance genes (Al-Mssallem et al., 2013). These SNP desserts can be embattled to develop resistance strategies to cope with significant biotic and abiotic stresses in the date palms through the CRISPR/Cas9 system (Sattar et al., 2017). Overall, CRISPR/Cas9-based genome editing allows scientists to study gene expression and target multiple loci in the date palm genome (Sattar et al., 2021).

## 3 CRISPR-based genome editing systems in trees

### 3.1 The CRISPR/Cas9 and CRISPR/Cas12a system

The CRISPR/Cas system has various variants. The most often deployed CRISPR/Cas variants are Cas9 (type II) and Cas12a (type V) (Liu et al., 2020). In the CRISPR system, any target site proceeding with a specified protospacer-adjacent motif (PAM) sequence 5’ NGG can be cleaved using Cas9. Some specific PAM targeting loci can be variable within the genome, so it does not always do precise editing. Previously, the CRISPR/Cas12a was called CpF1 (CRISPR from *Prevotella* and *francisella1*). The Cas12a system is a single RNA-guided endonuclease lacking tracrRNA (tetra loop sequence). It utilizes a T-rich PAM sequence 5’ TTN (Zetsche et al., 2015). The Cas12a and novel Cas8a, Cas8b, Cas3, Cas10d (type I), Cas1, Cas2 Cas6 (type III) are known for precision editing under various PAM motifs (Cheng et al., 2017; Guan et al., 2017; Safari et al., 2019). The Cas9-based editing produces blunt ends, whereas Cas12a develops staggered ends and permits site-directed integration (Bandyopadhyay et al., 2020). The CRISPR/Cas9 and Cas12a systems are effective tools for plant genome editing. The CRISPR/Cas9 system would be more efficient and specific for palms (Tang et al., 2019; Yarra et al., 2020; Moradpour and Abdulah, 2020).

**Figure 2:**
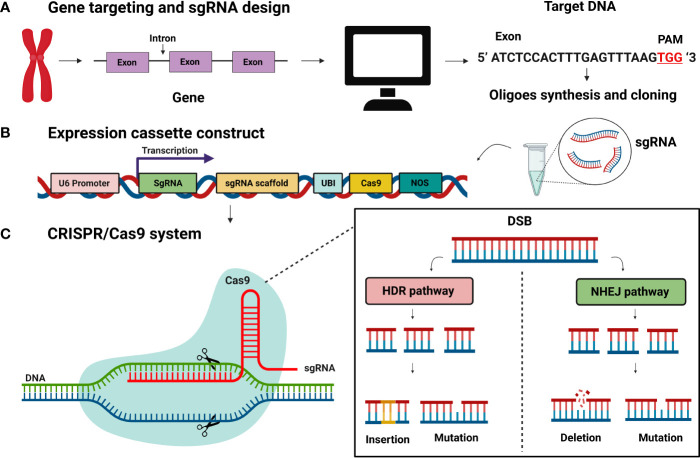
CRISPR/Cas9 system for genome editing. **(A)** Gene targeting and sgRNA construction. sgRNA expression constructs with 20bp target sequence **(B)** Expression construct for Cas9 and sgRNA **(C)** CRISPR/Cas9 system-based genome editing is DNA, DSBs (double-strand breaks) at target loci. DSBs induced genetic medication by two pathways: homology-directed mediated repair (HDR) and non-homologous end joining (NHEJ). The NHEJ process results in mutation and deletions.

The main feature of CRISPR/Cas9 is DNA double-strand breaks (DSBs) at target loci. The double-stranded breaks induce genetic modification by two pathways: homology-directed mediated repair (HDR) and non-homologous end joining (NHEJ) (**Figure 2**). The NHEJ pathway mediates DSB by inserting and deleting several nucleotides. The NHEJ can introduce the foreign sequence or remove the line in a precise manner, as well as knock out the gene. (Bernheim et al., 2017; Salsman and Dellaire, 2017). Aspects of the CRISPR/Cas9 system that are critical to the efficacy of gene editing are sgRNA efficiency, sgRNA the secondary structure (**Figure 2**), and target GC content (Ma et al., 2015). The CRISPR/Cas9 system has been widely used in commercial tree genetic improvement (Zafar et al., 2020; Huang et al., 2021). Numerous tree species have been edited successfully through the CRISPR/Cas9 technology, including, but not limited to, the cacao (*Theobroma cacao)* (Fister et al., 2018), apple (*Malus domestica*) (Nishitani et al., 2016), coffee (*Coffea canephora*) (Breitler et al., 2018), and citrus (*Carrizo citrange*) (Jia et al., 2017). However, the CRISPR/Cas9 system has some limitations for perennial tree genome editing which are summarized in (Triozzi et al., 2021).

The guide RNA in the CRISPR/Cas9 system is an assembly of tracrRNA (tetra loop sequence) and crRNA (target sequence). These RNAs streamline into a single RNA chimera known as single guide RNA (sgRNA) with dual tracrRNA and crRNA secondary structure used in genome editing (Uniyal et al., 2019). The sgRNA consists of a spacing sequence that complements the target DNA sequence to guide Cas9 proteins to the genomic target. The CRISPR/Cas9 system is based on the design of sgRNA to target the gene of interest. Cas9 nuclease uses sgRNA to target specific genomic sites using 20-22 nt (N_20-22_) custom nucleotides (Jain, 2015; Yue et al., 2020). The transcriptional activity of sgRNAs in plants is crucial for effectual genome editing *via* CRISPR/Cas9 (Jiang et al., 2013; Zhang et al., 2017a; Feng et al., 2018). Various online tools can predict the potential CRISPR target given an input sequence (**Supplementary Table 1**). Cas9 and sgRNA expression are essential for high mutation rates . The efficiency of sgRNA associated to a specific target gene can be identified by sequencing of the target genomic region (Bao et al., 2019).

The sgRNA transcription is directed by three RNA polymerase enzymes (Roeder and Rutter, 1969). RNA polymerase I (transcribes large rRNA), RNA polymerase II (transcribes mRNA, snRNA, and microRNA), and RNA polymerase III (transcribes small non-coding RNA) (Cramer et al., 2008).Commonly, the Cas9 protein is expressed through RNA polymerase II promoters such as cauliflower mosaic virus (CaMV35S) and ubiquitin (UBI) promoters (Feng et al., 2013; Mao et al., 2013). Furthermore, sgRNA expression can also be driven by RNA polymerase III (U6 and U3 promoters) (Haurwitz et al., 2010). Furthermore, non-coding RNAs, U6 small nuclear RNAs (snRNAs), perform intron splicing during the generation of mature mRNA in the eukaryotic cell (Long et al., 2018). While U3 snRNAs tangle in pre-rRNA dispensation (Venema et al., 2000). The U3 and U6 snRNA genes consist of a conserved TATA-box and an upstream sequence element (USE) (Li et al., 2007; Marz and Stadler, 2009). Rice (*Oryza sativa*) U6, OsU6a, OsU6b, and OsU6c are the most frequently used promoters for driving sgRNA in monocotyledons. The U6, AtU6-1, and AtU6-29 promoters preferably use in dicotyledons (Jiang et al., 2013; Li et al., 2014). The endogenous U6 promoter is used for successful genome editing in grapes and cotton (Long et al., 2018; Ren et al., 2021). Nevertheless, various studies have reported that species-specific U6 promoters are more efficient in driving sgRNA and enhancing editing efficiency (Long et al., 2018). Endogenous U6 and U3 promoters are rarely evaluated in palm genome editing because of the genome and selection complexities.

The Cas9 protein expression in monocots under endogenous plant promoters resulted in more on-target mutations than expression under the control of constitutive CaMV35S promoters (Ansari et al., 2020). Particularly, various studies have the direction to assess Cas9 expression under endogenous promoters, resulting in higher on-target mutation than CaMV35S (Haque et al., 2018; Long et al., 2018). In plants, sgRNA expression regulates by U6 and U3 promoters with a definite transcription start site (Ma et al., 2015; Hao et al., 2020). Transcripts formed under the control of eukaryotic U3 and U6 promoters commonly start with A and G at the 5’ end of the mature sgRNA with a N_19_ or N_20_ sequence followed by a protospacer-adjacent motif (PAM). The guide sequences were 19-nt N_19_ or 20-nt N_20,_ highly complementary to the target site. The Cas9/sgRNA complex binds a 20bp target sequence, then attaches to PAM, creating cleavage for DSB (Xie and Yang, 2013). Furthermore, scientists have achieved PAM-free Cas9 variations called SpG from *Streptococcus pyogenes* to expand its usefulness in genome sites with low GC content (Walton et al., 2020). These tools will contribute to the application of new biotechnologies. Despite its extensive application in crop biotechnology, there are a few challenges, like optimization of the role of Cas9 as well as reducing off-target rates (Ansari et al., 2020). An efficient CRISPR/Cas9 system for palm trees (coconut, oil palm, and date palm) is not easy to develop. Identifying the U6 promoter in the coconut oil palm and date palm genome databases is time-consuming to select the optimal one because the transformation system is complicated and laborious.

### 3.2 Tissue-specific Cas9 system

Since late 2019, tissue-specific genome editing has been reported (Ali et al., 2020). In recently registered tissue-specific genome editing techniques, tissue-specific promoters have a significant prospect for crop improvement by regulating the desired tissue or organ (Lei et al., 2021). The use of tissue-specific promoters gain a position in functional genomics due to its capacity to efficiently drive targeted gene expression in specific tissues. The choice of promoters and the sgRNA/Cas9 complex is essential for achieving the desired genome editing by the CRISPR/Cas9 system. The use of promoters in tissue-specific genome editing can help with conditional genetic modification, regulation of target gene expression, and potential induced mutation by favoring particular cellular and inducible sgRNA production. The studies on tissue-specific genome editing in various crops show its versatility. At the same time, in the case of the palm family, there is a need to set the groundwork for the optimization of tissue-specific GE experiments. Tissue-specific genome editing can effectively reduce the activity of the off-targets in the palm, which is not efficiently predicted and confirmed by *in-silico* predictions. Readers may get more detailed information about tissue-specific genome editing in a recently published review (Singha et al., 2022).

### 3.3 The ribozyme-mediated CRISPR/Cas9 system

Ribozymes are catalytic RNA molecules that catalyze reactions using sequence-specific interactions inside the RNA molecule (Serganov and Patel, 2007). The ribozyme-mediated CRISPR/Cas9 system significantly advances GE through pol II promoters (Lee et al., 2016). The ribozymes (hammerhead-type and the hepatitis delta virus) are added at the end of the sgRNA. The self-cleavage property of ribozyme disrupts the covalent link of RNA and separates it (Ferré-D'amaré et al., 1998; Gao and Zhao, 2014). Applying a ribozyme-based CRISPR/Cas9 system eases promoter selection for palms. The use of ribozyme-CRISPR/Cas9 technology expands the types of promoters available to the CRISPR/Cas9 system, allowing it to employ pol II promoters other than U6 and U3 (He et al., 2017b). In a recent study, a ribozyme-mediated CRISPR vector was constructed and transformed into hairy roots of pyrethrum to edit the *TcEbFS* (*E-B-farnesene synthase*) gene (Li et al., 2022). This system facilitates editing of a specific organ or a particular development site. The transgenic root (root hairs) line had a DNA base mutated at the editing target site. Overall, ribozyme-CRISPR/Cas9 improves the capacity of sgRNA to be transcribed. The ribozyme-CRISPR/Cas9 system can use RNA polymerase II-dependent promoters with the potential to express in specific target tissues (Li et al., 2022). Applying a ribozyme-mediated system decreases the difficulty of promoter selection in palm tree genome editing.

### 3.4 The base-editing system

CRISPR/Cas9 technology also represents a base-editing tool that emerges for robust and efficient genome editing. Base editing can be done to target sites by single nucleotide changes in DNA and RNA without cutting DSBs and HDR (Molla and Yang, 2019), resulting in the desired insertion/modification of the targeted DNA sequence. The HDR mode significantly involves point mutation, substitution, gene knock-in, and gene knockout (**Figure 2**) (Arnoult et al., 2017). The base-editing (BE) technology consists of Cas9 variants such as dCas9a and Cas9 nickase. These variants have a combination of cytosine and adenosine deaminases. Cytidine base editor (CBE) converts C-G bp into T-A bp. In contrast, adenine base editor (ABE) alters T-A bp into C-G bp (Gaudelli et al., 2017). Base editing would play a significant role in targeted random mutagenesis (Li et al., 2020) in the breeding of palms. This approach allows for genome editing that is simple, accurate, and efficient. However, this base editing approach has only been explored in model plants. It has yet to be used on commercial tree species like palms. The base editing system has some limitations, such as off-targets and low editing efficiency. This issue can be solved by finding a Cas protein with high specificity and efficiency.

### 3.5 The DNA-free genome editing system

DNA-free editing of plants is a new but emerging field that arose in 2015. In the past few years, a concern was raised about GMOs related to foreign DNA, which could limit the widespread use of CRISPR technology (Woo et al., 2015). CRISPR/Cas9 constructs are transferred into the plant *via* direct or indirect DNA delivery systems (Masani et al., 2014; Budiani et al., 2018; Wolter and Puchta, 2018; Darmawan et al., 2020; Yeap et al., 2021). The CRISPR/Cas9 construct has a good chance of being incorporated into the plant’s genome throughout this process (Zhang et al., 2016). The CRISPR/Cas9 technology optimizes precision breeding in several ways. The most important goals for optimization are to avoid transgene integration and to reduce off-target mutations (Metje-Sprink et al., 2019). This approach benefits trees with a long generation cycle (Malnoy et al., 2016). For CRISPR/Cas9 genome editing, reagents are delivered either through the introduction of Cas9 nuclease and guide RNA or by direct delivery of ribonucleoproteins (RNPs). CRISPR/Cas9 RNPs altogether avoided transgene integration and significantly reduced off-target mutation (Zhang et al., 2016; Liang et al., 2017). The RNPs delivered into protoplast and resulted in transgene-free mutant plants. Author Kim et al., 2017 (Kim et al., 2017) reported that Cpf1 could also be used for DNA-free genome editing, similar to the CRISPR/Cas9 system. Nevertheless, a limitation in plant regeneration from protoplast is challenging in trees, especially palm trees.

## 4 Methods of genetic transformation in palm trees

### 4.1 *Agrobacterium*-mediated transformation

**Figure 3:**
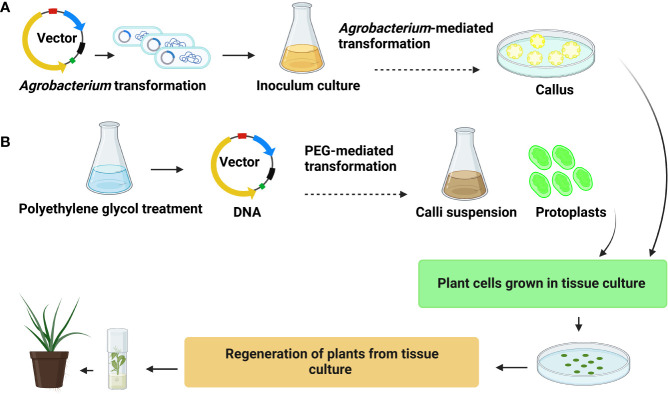
General representation **(A)** *Agrobacterium*-mediated transformation **(B)** polyethylene glycol (PEG)-mediated transformation.

*Agrobacterium tumefaciens* is a bacterium well-known for its ability to genetically engineer plant species (Ignacimuthu et al., 2000). However, the most successful and frequently used genetic transformation methods for palm trees use *Agrobacterium*-mediated transformation (**Table 2**; **Figure 3A**) (Blake, 1983). The *Agrobacterium*-mediated transformation successfully expressed transgene markers like the cowpea trypsin inhibitor (*CpTI*), *Bacillus thuringiensis*, and *PHB* in oil palm (Masli et al., 2009; Izawati et al., 2012). In oil palm, the 2-Deoxyglucose-6- phosphatase (*DOGR1*) gene was transformed *via Agrobacterium* strain LBA4404 in embryogenic calli (Dayang Izawati et al., 2015). In coconut, *Agrobacterium*-mediated transformation developed red and green fluorescent marker genes (Andrade-Torres et al., 2011). Date palm embryogenic callus transformation mediated by *Agrobacterium* strain AGL1 resulted in transient expression of *B-glucuronidase*. The gene transfer efficiency obtained in this study represents a sound basis for stable genetic transformation (Saker et al., 2009). A successful *Agrobacterium*-mediated transformation was reported in matured somatic embryos in date palm cultivar ‘Khalasah’ with LBA4404 harboring the PBI121 vector and uidA gene (Aslam et al., 2015). Notably, the developed embryo showed 47.5% transformation efficiency, and leaves displayed vigorous GUS activity. These established *Agrobacterium*-mediated transformation methods can be utilized for genome editing in palm trees (Aslam et al., 2015; Budiani et al., 2018).

**Table 2:** *Agrobacterium*-mediated transformation in palm trees.

Tree	Target	Explant	Strain	Infection time	Concentration	Reference
Date palm	GUS activity	Somatic embryos	LBA4404,	10 min	OD540 (0.520),	(Aslam et al., 2015)
Oil palm	CRISPR/Cas9	Callus	*Agrobacterium*	15 min	–	(Budiani et al., 2018)
Date palm	GUS activity	Embryogenic callus	AGL1	2 h	OD600 (1-1.5)	(Saker et al., 2009)
Coconut	Fluorescent proteins	Embryogenic calli	*Agrobacterium*	20 min	–	(Andrade-Torres et al., 2011)
Oil palm	*DOGR1*	Embryogenic calli	LBA4404	2 h	OD600 (0.2-0.4)	(Dayang Izawati et al., 2015)

### 4.2 PEG-mediated transformation

The application of genome editing in palm trees has to overcome certain biological and regulatory constraints. However, particle bombardment and polyethylene glycol (PEG)-based transformations can directly transfer sgRNA into the plant genome (Wolter and Puchta, 2018). A PEG-mediated transformation protocol has been established for oil palm protoplast transformation (**Figure 3B**). A previous study (Masani et al., 2022) stated that protoplast isolated from 3-month-old suspension calli were collected 14 days after culture. The protoplast is transformed with CFDV-hrGFP plasmid using a solution of PEG-3500 in magnesium chloride (MgCl_2_). PEG transfection requires optimizing conditions, such as concentration of DNA, PEG, MgCl_2_, and heat shock conditions. Transformation of oil palm protoplast *via* PEG-mediated transformation is promising (Masani et al., 2013). More than 20% transfection efficiency was achieved. Similarly, coconut protoplast was transformed by the pCAMBIA300s vector with the *CnMADS1* gene *via* PEG-mediated transformation. (Sun et al., 2020). Moreover, PEG-mediated transformation can assess the GE efficiency of different target genes by CRISPR/Cas9 technology. However, the difficulty in regenerating the transgenic plant from protoplast limits its application in trees (Masani et al., 2013).

### 4.3 Particle bombardment-mediated transformation

**Figure 4:**
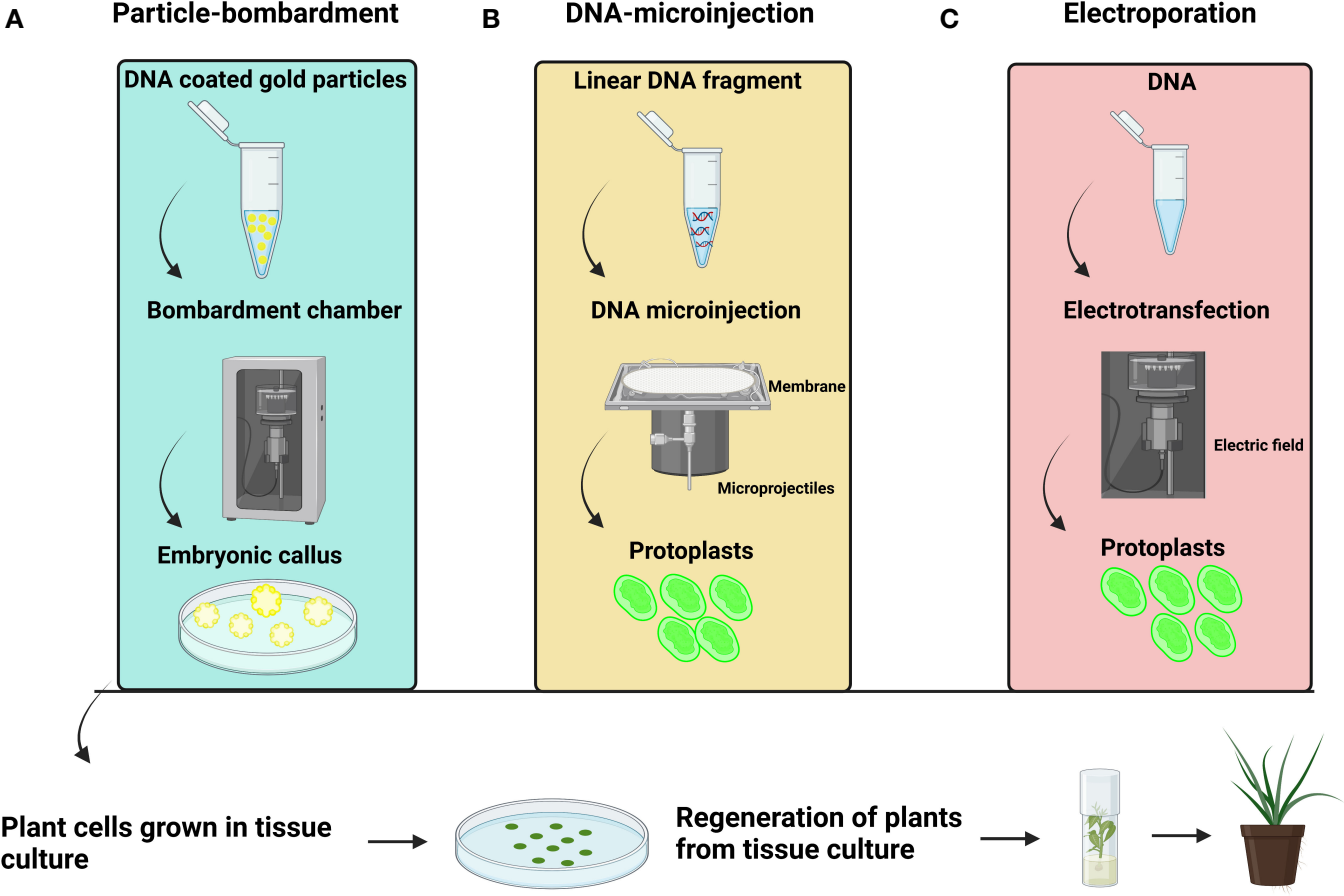
General representation **(A)** Particle bombardment-mediated transformation **(B)** DNA microinjection-mediated transformation **(C)** Electroporation-mediated transformation.

In plants, particle bombardment is widely used to deliver DNA directly into a plant cell (**Figure 4A**). Particle bombardment-mediated transformation is used for stable gene transfer into oil palm (**Table 3**) (Parveez and Christou, 1998). The use of suitable selectable markers and promoters during particle bombardment requires the development of physical and biological parameters during transformation. Usually, particle bombardment has been used to create herbicide glufosinate resistant transgenic oil palm (Parveez and Christou, 1998). Particles bombarded with genes related to fatty acid biosynthesis on embryonic calli of oil palm accumulate high oleic acid content (Kadir, 2003). Using biolistic particle bombardment into zygotic embryos, the newest study in oil palm attained 5-19.5% transformation efficiency, with up to 87% of embryos regenerating into shoots by direct embryogenesis (Yeap et al., 2021). It has a higher efficiency than *Agrobacterium*-mediated transformation, which has increased its application in palm trees. The efficiency of particle bombardment has been reported in Estamaran date palm cultivars the pCAMBIA3301 vector harboring the uidA gene under the CaMV35S promoter to transiently transform embryogenic callus (Mousavi et al., 2014a; Mousavi et al., 2014b). Particle bombardment-mediated transformation is a promising technique for GE in palm trees.

**Table 3:** Particle bombardment-mediated transformation in palm trees.

Tree	Explant	Construct	Instrument	Regeneration frequency	Reference
Date palm	Somatic embryos	Plasmid DNA	PDS1000/He	Yes	(Mousavi et al., 2014b)
Date palm	Embryogenic callus	DNA	PDS1000/He	No	(Mousavi et al., 2017)
Oil palm	Immature embryo	CRISPR	PDS-1000/He	Yes	(Yeap et al., 2021)
Oil palm	Embryonic callus	Plasmid DNA	Electric Discharge Device	Yes	(Parveez and Christou, 1998)
Oil palm	Embryonic callus	DNA	–	Yes	(Kadir, 2003)
Oil palm	Embryonic calli	Plasmid DNA	PDS-1000/He	Yes	(Bahariah et al., 2013)
Oil palm	Immature embryo	Plasmid DNA	PDS 1000/He	Yes	(Abdullah et al., 2005)

### 4.4 DNA microinjection-mediated transformation

A DNA microinjection-mediated transformation is also used in oil palm protoplast (Masani et al., 2014). The researchers utilized a 1% alginate layer to immobilize protoplasts for a successful transformation. Immobilized protoplasts were kept at 28°C for three days in the dark before being microinjected with the linear CFDV-hrGFP fragment (**Figure 4B**). After 72 hours of microinjection, they observed GFP expression. DNA microinjection-mediated transformation showed 47.6% transfection efficiency with 5µL of DNA (100ng/µL). The results showed that 14% of the injected protoplasts developed into micro calli that displayed GFP signals after six months.

### 4.5 Electroporation-mediated transformation

The electroporation method is used to identify the mutation rate and sgRNA efficiency (**Figure 4C**). The electroporation of Cas9 and sgRNA constructs resulted in a cleavage frequency of up to 25.49% in oil palm protoplasts (Liu et al., 2016; Yeap et al., 2021). Using plant tissue culture to enhance and maintain genetic transformation has become a tool for scientists those involved in enhancement of agricultural production. Electroporation substantially impacted callus growth rates (Darmawan et al., 2020). The pCAMBIA1303 with the *GUS* gene under the CaMV35S promoter can successfully transform into oil palm calli (Darmawan et al., 2020). Electric field strengths of 250, 500, 750, 1,000, and 1,250 V/cm has used for oil palm calli electroporation. All studied electric field strength treatments delivered gene detection in surviving calli, with 250V/cm electroporation resulting in the greatest calli growth rates.

## 5 Potential application of CRISPR/Cas9 system in palm trees

### 5.1 Genome-wide construction of mutant libraries

**Figure 5:**
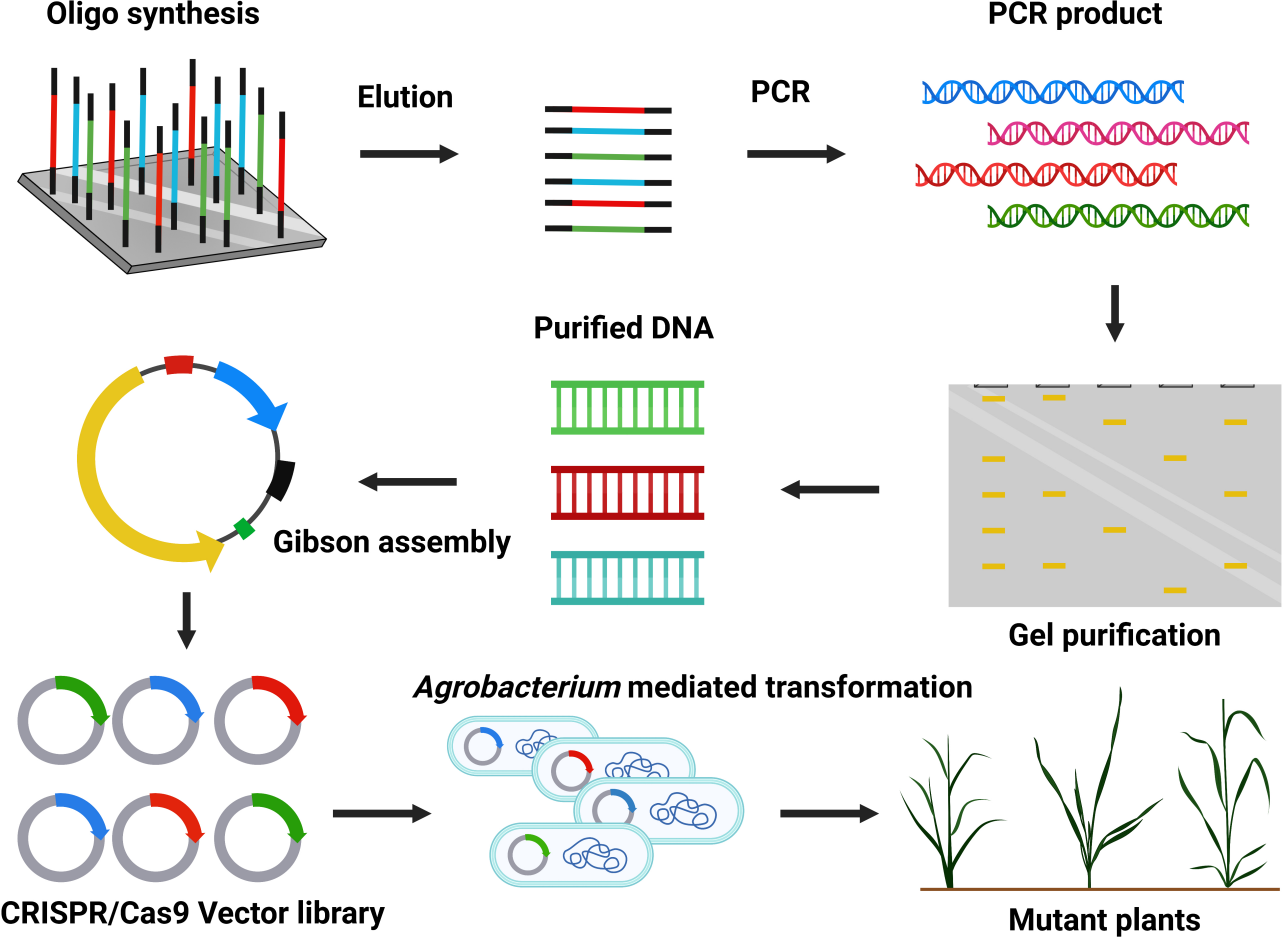
Construction of CRISPR/Cas9-mediated mutant library.

Large-scale mutant production at the genome-wide level is fundamental for functional genomics (**Figure 5**) (Lian et al., 2019). CRISPR/Cas9 technology holds excellent hope for genetic improvement in commercial crops. Previously, many mutants were formed by traditional methods (radiation and chemicals) and contributed to basic plant research. CRISPR/Cas9 technology can efficiently modify the genome and is used to generate plant mutant libraries for high throughput screening. The sgRNA can quickly be produced on a large scale by array-based synthesis of oligonucleotide libraries for multiplex genomic targeting (Peterson et al., 2016). Over time, only a few studies have developed a genome-scale mutant library *via* CRISPR/Cas9. Large-scale mutant libraries are successfully constructed in rice (*Oryza sativa*) (Meng et al., 2017), *Arabidopsis* (Peterson et al., 2016), and tomato (*Solanum lycopersicum*) (Jacobs et al., 2017). High expression of Cas9-sgRNA can increase off-target activity, so choosing a suitable promoter is crucial (Hsu et al., 2013; Pattanayak et al., 2013). CRISPR/Cas9 mutant library construction has not been generated in coconut, date palm, or oil palm trees. Recently, researchers have been interested in facilitating the genome-scale mutagenesis and production of transgene-free genome-edited palms. At CRI-CATAS, our group is currently constructing a genome-scale CRISPR/Cas9 mutant library with desired traits related to high fatty acid biosynthesis, seed development, disease resistance, and abiotic stress tolerance (**Figure 5**).

### 5.2 Identification and modification of genes related to fatty acids in palm trees

In recent years, vegetable oil consumption has expanded dramatically (FAO, 2018). As a result, identifying the oil biosynthesis-related genes and using them for genetic improvement is an effective strategy to increase edible oil production. The application of CRISPR/Cas9 system can potentially modify fatty acid biosynthesis and oil yield traits. The fatty acid/oil biosynthesis process is far more complex than a simple linear pathway and is controlled by multiple genes (Zhang et al., 2018). The *WRI1 (WRINKLED1)* and *DGAT* (diacylglycerol acyltransferase 1) genes are widely involved in lipid synthesis in seeds and leaves (Vanhercke et al., 2013). At the same time, *PDAT* (phospholipid diacylglycerol acyltransferase) is responsible for the last step of triacylglycerol synthesis. The enzyme SAD (stearoyl-CoA 9-desaturase) produces oleic acid by dehydrating stearic acid. The oleate desaturase enzyme converts oleic acid to linolenic acid and encodes by the *FAD2* (*fatty acid desaturase 2*) gene. The *FAD2* gene is responsible for polyunsaturated lipid synthesis in developing seeds (Zhang et al., 2012). Silencing *FAD2* caused a high oleic acid content in *Brassica napus* and *Arabidopsis* (Stoutjesdijk et al., 2000; Baoming et al., 2011). Editing of *OsFAD2* genes has been reported in rice (Bahariah et al., 2021). The *FAD2* and *PAT* (palmitoyl-ACP-thioesterase) genes are involved in the regulation of fatty acid composition. In plants, *PAT* is required for chain termination during *de novo* fatty acid synthesis and carbon flux channeling between two lipid biosynthesis pathways (Jones et al., 1995). Genome editing reduced the activity of *FAD2* and *PAT*. These genes enhanced oleic acid content by more than 70%. Further, FAD2 and PAT decreased linolenic acid and polyunsaturated fatty acids (Bahariah et al., 2021).

**Figure 6:**
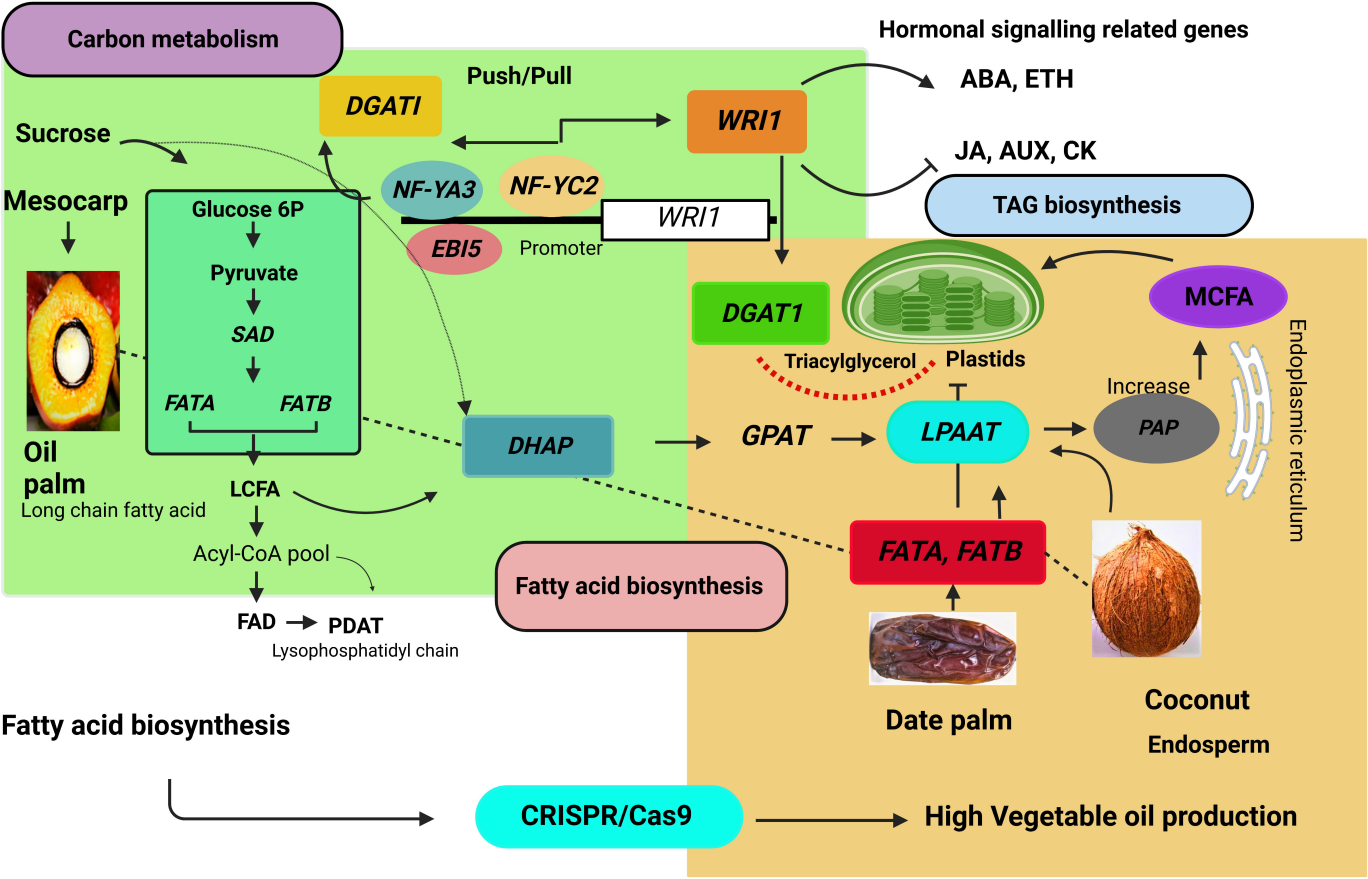
Fatty acid biosynthesis pathway in the palm tree. *DGAT1*, *diacylglycerol acyltransferase 1*; *WRI1, WRINKLED*; MCFA, medium chain fatty acid; LCFA, Long-chain fatty acid, *LPAAT*, *lysophosphatidic acid acyltransferase*; *FATA*, *acyl-CoA thioesterase A*; *FATB*, *acyl-CoA thioesterase B*; *EBI5*, *ABA INSENTIVE 5*; *NF-YA3*, *NUCLEAR FACTOR Y subunit A3*; *NF-YC2, NUCLEAR FACTOR Y* subunit *C2*; *WRKY40*, *WRKY transcription factor 40*; *WRKY2*, *WRKY transcription factor 2*. *SAD, stearoyl-CoA 9-desaturase* ***;*** *FAD*, *fatty acid desaturase; PDAT, phospholipids diacyleglycerol transferase; GPAT, glycerol-3-phosphate; PAP*, *phosphatic acid phosphohydrolase.*

High palmitic acid content makes palm oil unhealthy for human consumption. Thus, CRISPR/Cas9 offers several advantages to override genetic changes in high fatty acid and low palmitic acid content. The *EgFATB1* (Acyl-ACP thioesterase B) genes regulate palmitic acid concentrations and fatty acid composition in the mesocarp. Additionally, the *FatB* mutant showed regulation in palmitic acid content in oil palm (Xia et al., 2019). The *CnFATA* (acyl-ACP thioesterase class A) stimulates the TAG profile comprised of saturated fatty acid synthesis (FAs) (**Figure 6**) (Reynolds et al., 2019). Among five *CnFATB* genes, *CnFatB3* is expressed explicitly in the endosperm. TAG is the main component of vegetable oil, consisting of glycerol esterified with three fatty acids (Dussert et al., 2013). In contrast, *FAT genes* have been one of the attractive targets of genome editing in the commercial production of oil.

*EgNF-YA3*, *EgNF-YC2*, and *EgABI5* bind to the *EgWRI1* transcription factor promoter and regulate *EgWRI1* expression. Ectopic expression of *WRI1* controls the metabolic processes that might influence fatty acid accumulation by increasing TAG content in Arabidopsis seedlings (Cernac and Benning, 2004). Moreover, the *CnWRI1* gene synthesized high palmitic and linolenic acid content and decreased oleic acid content in *Arabidopsis* seeds (Reynolds et al., 2015). *WRI1* gene and palmitoyl-acyl carrier protein (ACP) thioesterase (FATB/PATE) co-expression increases total oil content. Furthermore, this expression network expanded to other regulatory pathways, such as sugar metabolism, starch metabolism, and glycolysis (Ting et al., 2020). *EgNF-YA3*, *EgNF-YC2*, and *EgABl5* activate *EgWRI1*. Overexpression of *EgWRI1* in *Arabidopsis* regulates ethylene-responsive transcription factors and abscisic acid-responsive element binding factors. The *EgWRKY4*0 and *EgWRKY2* transcription factors bind to the W-Box EBI5, while *EgWRKY40* negatively regulates *EgEBI5* and represses fatty acid biosynthesis (Yeap et al., 2017). The *EgGDSL* gene stimulates oil biosynthesis (Zhang et al., 2018). CRISPR/Cas9-based genome editing may be able to edit these binding sites, elucidate the exact role, and expose the oil yield-associated expression at the transcriptional level in the oil palm. The biochemical pathway leading to palm oil synthesis involves multiple subcellular organelles, requiring extensive lipid trafficking, such as mesocarp plastidial FA and endoplasmic reticulum-based ER-TAG. The FA controls the biosynthesis of storage oil, regulated by the *WRI1* gene encoded by the *AP2 (APETALA)* ethylene-responsive element-binding protein transcription factor (Dussert et al., 2013).

Expression of *DGAT*, *DGAT2A*, and *DGAT1-2* increases overall oil content in *Arabidopsis*, maize, and soybean. DGAT (diacylglycerol acyltransferase) enzyme-catalyzed acylation of DAG impacts fatty acid accumulation by TAG synthesis (Kumar et al., 2016). Moreover, *EgNF-YA3*, *EgNF-YC3*, and *EgABl5* transcriptionally activate *EgDGAT1*. A synergistic relationship of push/pull found between DGAT1 and WRI1 increases carbon flux in TAG biosynthesis (**Figure 6**) (Vanhercke et al., 2013). Lysophosphatidic acid acyltransferase (LPAAT) is involved in DAG production *via* the Kennedy route (Bernerth and Frentzen, 1990). Coconut oil is stored in solid endosperm and contains 47.8%-50.5% acid content as lauric acid. Acyl-acyl carrier protein thioesterases and lysophosphatidic acid acyltransferase enzymes are critical components in fatty acid accumulation in coconut oil. The study of coconut embryogenic genes and other gene encoding factors involved in lipid metabolism, such as the B3 domain transcription factor family (Kim et al., 2014). Coconut and oil palm contains 50% lauric acid in their endosperm. Gene expression and function are highly conserved in lipid metabolism pathways. It is also worth considering that successful CRISPR/Cas9-based genetic improvement in palm trees will enhance the commercial production of vegetable oil.

## 6 Limitations in palm trees CRISPR-based genome editing

### 6.1 Genome complexities and heterozygosity

Despite the broad application of CRISPR/Cas9-based genome editing, it still poses some limitations in palm trees. Plant models used for genome editing *via* CRISPR/Cas9 are mostly highly inbred. Due to the vast and complex genome, high rate of heterozygosity, and outcrossing, using CRISPR/Cas9 in palm genome editing can be challenging (Jubrael et al., 2005; Sattar et al., 2017). Identifying the genetic foundation of desirable traits is still a time-consuming process that requires a combination of forwarding and reversing genetic techniques and whole-genome sequencing utilizing next-generation sequencing techniques. Genome editing in date palms has some limitations, as genomes of outcrossing species have high allelic heterozygosity, polymorphism, and genetic instability (Jubrael et al., 2005). High allelic heterozygosity and sequence polymorphism may affect genome editing in woody perennial trees (Tsai and Xue, 2015). The oil palm genome is heterogeneous with low genetic variation, which is a significant constraint in genetic improvement (Babu et al., 2021). Coconut has a high heterozygosity rate, making genome editing an effort and a challenging process (Rajesh et al., 2021). For the implication of the CRISPR/Cas9 system in palm trees, prior knowledge of genetic variation and SNPs are required. On the other hand, the shortage of data on the best expression cassettes for expressing constructed nuclease, low transformation efficiency, slower growth rate, and trouble isolating GE clonal plants are those points that should be kept in mind (Limera et al., 2017).

### 6.2 Genetic transformation

The CRISPR/Cas9 system in palm trees has been hampered by a lack of an effective genetic transformation techniques, plant regeneration procedure, and *in vitro* testing tools. Several transformation methods to deliver the CRISPR system into plants have been tried. However, to overcome the constraint, comprehensive genome engineering technologies must be developed to make them more practical and beneficial for the palm family because the palm family has very low transformation efficiency compared to other plants (Cutter and Wilson, 1954; Blake, 1983; Teixeira et al., 1995). In oil palm, *Agrobacterium*-mediated transformation efficiency is 0.7% (Masli et al., 2009; Izawati et al., 2012), whereas 1.5% for particle bombardment (Parveez et al., 2000) and 14% for protoplast DNA microinjection (Masani et al., 2014). Protoplast transformations are commonly applied as a transient expression system to assess the CRISPR/Cas9 system’s viability. However, due to limits and inheritable mutations, it is hard to generate plants from protoplast cultures. Oil palm genome editing for genetic improvement *via* seed propagation is time-consuming, selection cycles run for more than ten years, and there is a lot of variation among oil palm hybrids (Weckx et al., 2019). An inefficient plantlet regeneration system in coconut is a significant limitation in genome editing due to slow growth and a lack of vigor (Nguyen et al., 2015). The ribozyme-mediated CRISPR/Cas9 system improved the CRISPR/Cas9 system through highly effective, rapid, and simple *Agrobacterium* ribozyme-mediated transformation in hairy roots (Triozzi et al., 2021). For palm trees, transformation and regeneration remain major bottlenecks after genome editing. However, regeneration of transformed tissues, optimization of selection pressure, tediousness, time-consuming procedures, and low transformation efficiency remain major challenges.

### 6.3 The CRISPR/Cas9 vector construction and sgRNA design

The CRISPR/Cas9 vector construct is one of the crucial elements for its effective performance. Additionally, when more genes responsible for oil palm agro-economical features are discovered, editing at a single locus in the genome may not produce the desired result if numerous genes rule the trait (Aprilyanto et al., 2019). Moreover, the critical step in genome editing is the selection of sgRNA (target region in the genome). Generally, in palm trees, the choice of the target region in the genome may be comprised of polymorphism, off-targets, the presence of introns, and SNPs (Sattar et al., 2017; Sattar et al., 2021). Date palm cultivars’ whole genome sequencing revealed intra- and inter-varietal SNPs not only in the intergenic region but also in the coding region (Al-Mssallem et al., 2013; Sabir et al., 2014). As a result, it is necessary to identify the sgRNA features that optimize Cas9 nuclease activity. Various web-based tools for sgRNA design in plants have restricted the availability of palm species databases (**Supplementary Table 1**). It could be due to the scarcity and discovery of palm family genomes on the available resources. In the CRISPR/Cas9 system, there is limited choice for promoters complementary to the target sequence, which guides Cas9 to generate DSBs at the target site (He et al., 2017a). In addition, several key factors can also affect the CRISPR/Cas9-based genome editing in palm trees. These may include the GC content and size of sgRNA, Cas9 protein, and co-expression of sgRNA during the pairing of sgRNA with the target sequence (Fu et al., 2014).

### 6.4 Off-targets

There is a desperate need to rectify all possible irregularities in CRISPR/Cas9-mediated genome editing to minimize off-targets. Whole-genome sequencing of coconut, oil palm, and date palm showed intra- and inter-varietal SNPs in both the intergenic and coding regions. Currently, no method can precisely predict off-target sites (Huang et al., 2022).

## 7 Future perspective

The increasing climatic changes, biotic factors, and human population boom have left no choice for biotechnologists except to develop genetic tolerance for the sustainable production of commercial palms. CRISPR-based targeted GE in perennial trees and woody vines like oil palm, apple, grape, kiwifruit, poplar, sweet orange, and grapefruit have been done in the past (**Table 1**). There are many applications of CRISPR in plant biotechnology; like sex determination (Sattar et al., 2017). Applying next-generation DNA-free CRISPR/Cas9 approaches such as CRISPR/Cas9 ribonucleoproteins (RNPs) may offer a reasonable solution to address current GMO regulations in certain countries (Kanchiswamy, 2016; Lu et al., 2017). CRISPR-based GE can also be explored to restrain the acute diseases and pests of the palm family. Moreover, the classical mutagenic techniques are not necessarily suitable for the inactivation of every gene under study due to the inconstant nature of gene integration, which could end up in the appearance of transgenes (Khatodia et al., 2016). Such randomly induced mutagenesis has the severe drawback of an enormous background mutation load (Braatz et al., 2017). However, the comprehensive mutagenesis made only possible by the CRISPR/Cas9 technology would allow researchers to target several loci in the in palms genome and to analyze the expression of such fixed genes. The site-specific gene insertion, specificity in targeted mutation, and controlled genetic manipulations can make CRISPR/Cas9 a novel tool for commercial palms GE in the future.

## 8 Conclusion

**Figure 7:**
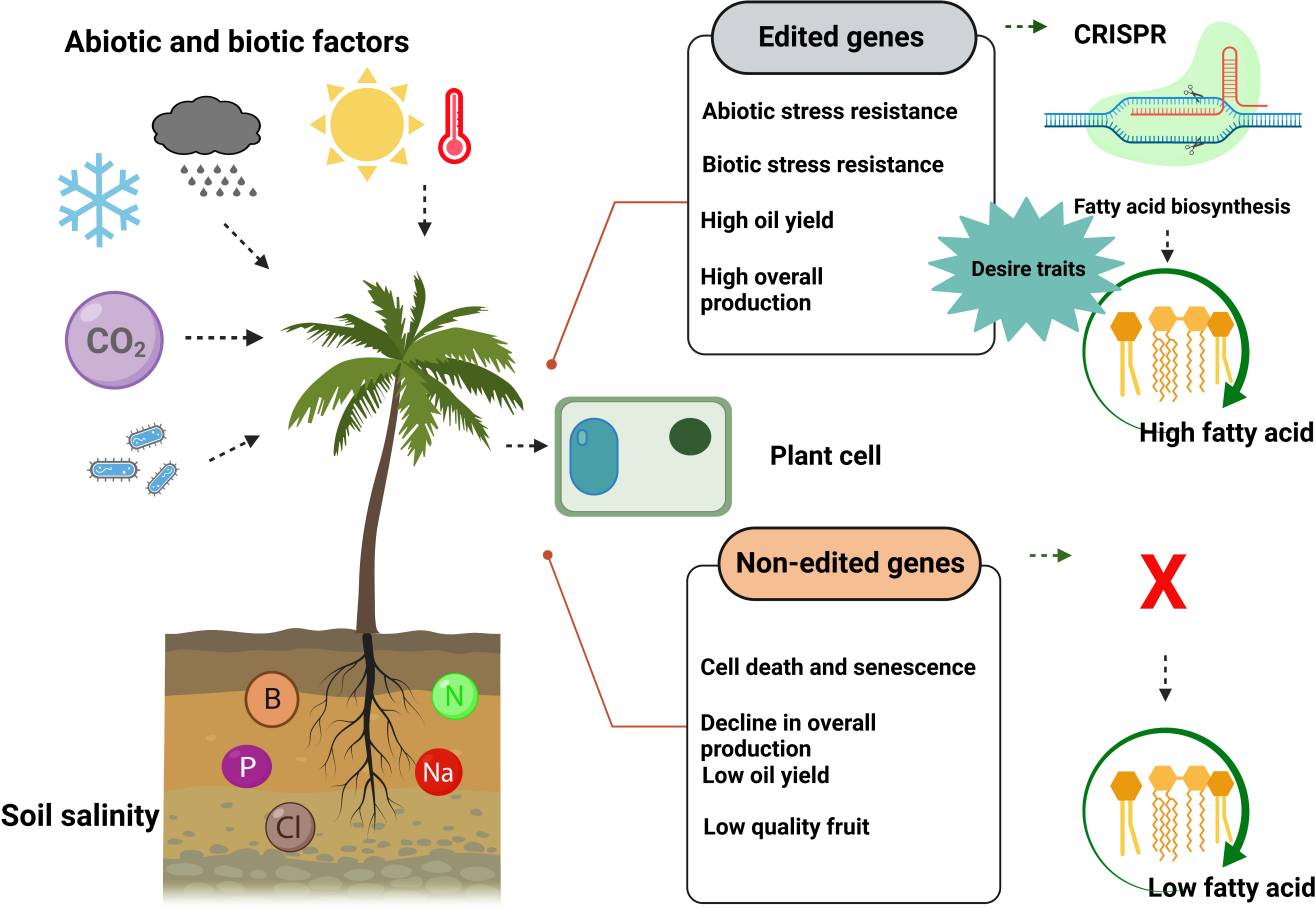
CRISPR/Cas9-mediated genome editing of palm trees produced more fatty acids and showed resistance against biotic and abiotic stresses. In contrast, non-edited palm trees produce less fatty acid (vegetable oil) and are unable to resist biotic and abiotic stresses.

CRISPR-based genome editing could play an integral role in achieving climate resilience, high yielding, and stress tolerance-related genetic traits in commercial palms (**Figure 7**). CRISPR has become one of the prominent and widely utilized biotechnology for GE in various plant species with many success stories in the past and holds massive potential in precision crop breeding, with some technical limitations. Despite CRISPR/Cas9’s massive potential, GE in palms can have specific bottle-necks. Genome data mining, selection of target, transformation method, high allelic heterozygosity, outcrossing, sequence polymorphism, off-targets, and the large genome size of palm trees are the real challenges in the middle. Before claiming a big win in the case of palms, we must address some hot questions; Are consumers willing to choose gene-edited products such as vegetable oil? Is this technology going to be cost-effective? Although CRISPR/Cas9-mediated GE could reduce customers’ apprehensions due to its non-GMO nature, at the same time, spreading awareness about CRISPR-based GE in palms is still an essential task to do. We have touched the tip of an iceberg, as genome editing and genetic improvement are massive fields yet to be explored in palm trees. In the end, by considering the above-discussed reports of GE in palms, it is possible to predict that CRISPR-based GE can be promoted to obtain sustainable production of commercial palms with the desired genetic traits.

## Author contributions

Conceptualization, FK and YW; Writing-original draft preparation, FK; Writing-Review & Editing, FG, DZ, PS, ZL and YMH; Supervision, YW. All authors have read and agreed to the published version of the manuscript.

## References

[B1] Abdullah S. N. A. Azzeme A. M. Ebrahimi M. Ariff E. A. K. E. Hanifiah F. H. A. (2017). “Transcription factors associated with abiotic stress and fruit development in oil palm,” In Crop improvement (Cham: Springer), 71–99.

[B2] Abdullah R. Zainal A. Yew Heng W. Chui Li L. Chee Beng Y. Mei Phing L.. (2005). Immature embryo: A useful tool for oil palm (Elaeis guineensis jacq.) genetic transformation studies. Electron. J. Biotechnol. 8, 24–34. doi: 10.2225/vol8-issue1-fulltext-1

[B3] Adam D. (2021). How far will global population rise? researchers can’t agree. Nature 597, 462–465. doi: 10.1038/d41586-021-02522-6 34548645

[B4] Adkins S. W. Foale M. A Samosir Y. M. S. (2006). Coconut revival: New possibilities for the'Tree of life'. Proceedings of the International Coconut Forum held in Cairns, Australia 125, 22–24. Available at: hdl.handle.net/102.100.100/177590?index=1.

[B5] Ali M. A. Al-Hattab T. A. Al-Hydary I. A. (2015). Extraction of date palm seed oil (Phoenix dactylifera) by soxhlet apparatus. Int. J. Adv. Eng. Technol. 8, 261.

[B6] Ali Z. Mahfouz M. M. Mansoor S. (2020). CRISPR-TSKO: A tool for tissue-specific genome editing in plants. Trends Plant Sci. 25, 123–126. doi: 10.1016/j.tplants.2019.12.002 31859038

[B7] Aljohi H. A. Liu W. Lin Q. Zhao Y. Zeng J. Alamer A.. (2016). Complete sequence and analysis of coconut palm (Cocos nucifera) mitochondrial genome. PloS One 11, e0163990. doi: 10.1371/journal.pone.0163990 27736909 PMC5063475

[B8] Al-Mssallem I. S. Hu S. Zhang X. Lin Q. Liu W. Tan J.. (2013). Genome sequence of the date palm phoenix dactylifera l. Nat. Commun. 4, 1–9. doi: 10.1038/ncomms3274 PMC3741641 23917264

[B9] Andrade-Torres A. Oropeza C. Sáenz L. González-Estrada T. Ramírez-Benítez J. Becerril K.. (2011). Transient genetic transformation of embryogenic callus of Cocos nucifera. Biologia 66, 790–800. doi: 10.2478/s11756-011-0104-4

[B10] An Y. Geng Y. Yao J. Fu C. Lu M. Wang C.. (2020). Efficient genome editing in populus using CRISPR/Cas12a. Front. Plant Sci. 11, 593938. doi: 10.3389/fpls.2020.593938 33329659 PMC7720674

[B11] Ansari W. A. Chandanshive S. U. Bhatt V. Nadaf A. B. Vats S. Katara J. L.. (2020). Genome editing in cereals: Approaches, applications and challenges. Int. J. Mol. Sci. 21, 4040. doi: 10.3390/ijms21114040 32516948 PMC7312557

[B12] Aprilyanto V. Darmawan C. Utomo C. Liwang T. (2019). “Development of CRISPR/Cas9 plasmid for multiple sites genome editing in oil palm (Elaeis guineensis jacq.),” in AIP conference proceedings, vol. 2099. (AIP Publishing LLC), p. 020002.

[B13] Arnoult N. Correia A. Ma J. Merlo A. Garcia-Gomez S. Maric M.. (2017). Regulation of DNA repair pathway choice in s and G2 phases by the NHEJ inhibitor CYREN. Nature 549, 548–552. doi: 10.1038/nature24023 28959974 PMC5624508

[B14] Aslam J. Khan S. A. Azad M. A. K. (2015). Agrobacterium-mediated genetic transformation of date palm (Phoenix dactylifera l.) cultivar" khalasah" via somatic embryogenesis. Plant Sci. Today 2, 93–101. doi: 10.14719/pst.2015.2.3.119

[B15] Babu B. K. Mathur R. Anitha P. Ravichandran G. Bhagya H. (2021). Phenomics, genomics of oil palm (Elaeis guineensis jacq.): Way forward for making sustainable and high yielding quality oil palm. Physiol. Mol. Biol. Plants 27, 587–604. doi: 10.1007/s12298-021-00964-w 33854286 PMC7981377

[B16] Bahariah B. Masani M. Y. A. Abd Rasid O. Parveez G. K. A. (2021). Multiplex CRISPR/Cas9-mediated genome editing of the FAD2 gene in rice: A model genome editing system for oil palm. J. Genet. Eng. Biotechnol. 19, 1–13. doi: 10.1186/s43141-021-00185-4 34115267 PMC8196110

[B17] Bahariah B. Parveez G. K. A. Masani M. Y. A. Masura S. S. Khalid N. Othman R. Y. (2013). Biolistic transformation of oil palm using the phosphomannose isomerase (pmi) gene as a positive selectable marker. Biocatal. Agric. Biotechnol. 2, 295–304. doi: 10.1016/j.bcab.2013.08.004

[B18] Bandyopadhyay A. Kancharla N. Javalkote V. S. Dasgupta S. Brutnell T. P. (2020). CRISPR-Cas12a (Cpf1): A versatile tool in the plant genome editing tool box for agricultural advancement. Front. Plant Sci. 11, 1589. doi: 10.3389/fpls.2020.584151 PMC7668199 33214794

[B19] Bao A. Burritt D. J. Chen H. Zhou X. Cao D. Tran L.-S. P. (2019). The CRISPR/Cas9 system and its applications in crop genome editing. Critic. Rev. Biotechnol. 39, 321–336. doi: 10.1080/07388551.2018.1554621 30646772

[B20] Baoming T. Sun D. Yuli L. Haiyan S. Hua L. Xin Z.. (2011). Analysis of the RNAi targeting FAD2 gene on oleic acid composition in transgenic plants of brassica napus. Afr. J. Microbiol. Res. 5, 817–822. doi: 10.5897/AJMR10.823

[B21] Bao A. Zhang C. Huang Y. Chen H. Zhou X. Cao D. (2020). Genome editing technology and application in soybean improvement. Oil Crop Sci. 5, 31–40. doi: 10.1016/j.ocsci.2020.03.001

[B22] Bekheet S. A. Hanafy M. (2011). “Towards sex determination of date palm,” in Date palm biotechnology (Dordrecht: Springer), 551–566.

[B23] Bernerth R. Frentzen M. (1990). Utilization of erucoyl-CoA by acyltransferases from developing seeds of brassica napus (L.) involved in triacylglycerol biosynthesis. Plant Sci. 67, 21–28. doi: 10.1016/0168-9452(90)90046-Q

[B24] Bernheim A. Calvo-Villamañán A. Basier C. Cui L. Rocha E. P. Touchon M.. (2017). Inhibition of NHEJ repair by type II-a CRISPR-cas systems in bacteria. Nat. Commun. 8, 1–9. doi: 10.1038/s41467-017-02350-1 29234047 PMC5727150

[B25] Blake J. (1983). “Tissue culture propagation of coconut, date and oil palm,” In Tissue culture of trees (Boston, MA: Springer), 29–50.

[B26] Braatz J. Harloff H.-J. Mascher M. Stein N. Himmelbach A. Jung C. (2017). CRISPR-Cas9 targeted mutagenesis leads to simultaneous modification of different homoeologous gene copies in polyploid oilseed rape (Brassica napus). Plant Physiol. 174, 935–942. doi: 10.1104/pp.17.00426 28584067 PMC5462057

[B27] Breitler J.-C. Dechamp E. Campa C. Zebral Rodrigues L. A. Guyot R. Marraccini P.. (2018). CRISPR/Cas9-mediated efficient targeted mutagenesis has the potential to accelerate the domestication of coffea canephora. Plant Cell Tissue Organ Cult. 134, 383–394. doi: 10.1007/s11240-018-1429-2

[B28] Budiani A. Putranto R. Riyadi I. Minarsih H. Faizah R. (2018). “Transformation of oil palm calli using CRISPR/Cas9 system: toward genome editing of oil palm,” In IOP Conference Series: Earth and Environmental Scienc, vol. 183. (IOP Publishing), 012003

[B29] Cernac A. Benning C. (2004). WRINKLED1 encodes an AP2/EREB domain protein involved in the control of storage compound biosynthesis in arabidopsis. Plant J. 40, 575–585. doi: 10.1111/j.1365-313X.2004.02235.x 15500472

[B30] Cheng F. Gong L. Zhao D. Yang H. Zhou J. Li M.. (2017). Harnessing the native type IB CRISPR-cas for genome editing in a polyploid archaeon. J. Genet. Genomics 44, 541–548. doi: 10.1016/j.jgg.2017.09.010 29169919

[B31] Comai L. (2005). The advantages and disadvantages of being polyploid. Nat. Rev. Genet. 6, 836–846. doi: 10.1038/nrg1711 16304599

[B32] Cramer P. Armache K.-J. Baumli S. Benkert S. Brueckner F. Buchen C.. (2008). Structure of eukaryotic RNA polymerases. Annu. Rev. Biophys. 37, 337–352. doi: 10.1146/annurev.biophys.37.032807.130008 18573085

[B33] Cutter J. V. Wilson K. S. (1954). Effect of coconut endosperm and other growth stimulants upon the development *in vitro* of embryos of Cocos nucifera. Bot. Gaz 115, 234–240. doi: 10.1086/335819

[B34] Darmawan C. Wiendi N. M. A. Utomo C. Liwang T. (2020). Electroporation-mediated genetic transformation of oil palm (Elaeis guineensis). J. Biodiversitas 21(8). doi: 10.13057/biodiv/d210839

[B35] Dayang Izawati A. M. Abdul Masani M. Y. Ismail I. Ghulam Kadir A. P. (2015). Evaluation on the effectiveness of 2-deoxyglucose-6-phosphate phosphatase (DOGR1) gene as a selectable marker for oil palm (Elaeis guineensis jacq.) embryogenic calli transformation mediated by agrobacterium tumefaciens. Front. Plant Sci. 6, 727. doi: 10.3389/fpls.2015.00727 26442041 PMC4585222

[B36] Dussert S. Guerin C. Andersson M. Joët T. Tranbarger T. J. Pizot M.. (2013). Comparative transcriptome analysis of three oil palm fruit and seed tissues that differ in oil content and fatty acid composition. Plant Physiol. 162, 1337–1358. doi: 10.1104/pp.113.220525 23735505 PMC3707537

[B37] FAO (2018). Food and agriculture organization of the united nations (Rome: FAOSTAT).

[B38] Feng C. Su H. Bai H. Wang R. Liu Y. Guo X.. (2018). High-efficiency genome editing using a dmc1 promoter-controlled CRISPR/Cas9 system in maize. Plant Biotechnol. J. 16, 1848–1857. doi: 10.1111/pbi.12920 29569825 PMC6181213

[B39] Feng Z. Zhang B. Ding W. Liu X. Yang D.-L. Wei P.. (2013). Efficient genome editing in plants using a CRISPR/Cas system. Cell Res. 23, 1229–1232. doi: 10.1038/cr.2013.114 23958582 PMC3790235

[B40] Ferré-D'amaré A. R. Zhou K. Doudna J. A. (1998). Crystal structure of a hepatitis delta virus ribozyme. Nature 395, 567–574. doi: 10.1038/26912 9783582

[B41] Fister A. S. Landherr L. Maximova S. N. Guiltinan M. J. (2018). Transient expression of CRISPR/Cas9 machinery targeting TcNPR3 enhances defense response in theobroma cacao. Front. Plant Sci. 9, 268. doi: 10.3389/fpls.2018.00268 29552023 PMC5841092

[B42] Fu Y. Sander J. D. Reyon D. Cascio V. M. Joung J. K. (2014). Improving CRISPR-cas nuclease specificity using truncated guide RNAs. Nat. Biotechnol. 32, 279–284. doi: 10.1038/nbt.2808 24463574 PMC3988262

[B43] Gao Y. Zhao Y. (2014). Self-processing of ribozyme-flanked RNAs into guide RNAs *in vitro* and *in vivo* for CRISPR-mediated genome editing. J. Integr. Plant Biol. 56, 343–349. doi: 10.1111/jipb.12152 24373158

[B44] Gaudelli N. M. Komor A. C. Rees H. A. Packer M. S. Badran A. H. Bryson D. I.. (2017). Programmable base editing of a• T to g• c in genomic DNA without DNA cleavage. Nature 551, 464–471. doi: 10.1038/nature24644 29160308 PMC5726555

[B45] Guan J. Wang W. Sun B. (2017). Chromosomal targeting by the type III-a CRISPR-cas system can reshape genomes in staphylococcus aureus. Msphere 2, e00403–e00417. doi: 10.1128/mSphere.00403-17 29152580 PMC5687920

[B46] Hao Y. Zong W. Zeng D. Han J. Chen S. Tang J.. (2020). Shortened snRNA promoters for efficient CRISPR/Cas-based multiplex genome editing in monocot plants. Sci. China Life Sci. 10, 933–935. doi: 10.1007/s11427-019-1612-6 31942685

[B47] Haque E. Taniguchi H. Hassan M. Bhowmik P. Karim M. R. Śmiech M.. (2018). Application of CRISPR/Cas9 genome editing technology for the improvement of crops cultivated in tropical climates: recent progress, prospects, and challenges. Front. Plant Sci. 9, 617. doi: 10.3389/fpls.2018.00617 29868073 PMC5952327

[B48] Haurwitz R. E. Jinek M. Wiedenheft B. Zhou K. Doudna J. A. (2010). Sequence-and structure-specific RNA processing by a CRISPR endonuclease. Science 329, 1355–1358. doi: 10.1126/science.1192272 20829488 PMC3133607

[B49] Hazzouri K. M. Flowers J. M. Visser H. J. Khierallah H. S. Rosas U. Pham G. M.. (2015). Whole genome re-sequencing of date palms yields insights into diversification of a fruit tree crop. Nat. Commun. 6, 1–11. doi: 10.1038/ncomms9824 PMC4667612 26549859

[B50] Hazzouri K. M. Gros-Balthazard M. Flowers J. M. Copetti D. Lemansour A. Lebrun M.. (2019). Genome-wide association mapping of date palm fruit traits. Nat. Commun. 10, 1–14. doi: 10.1038/s41467-019-12604-9 31615981 PMC6794320

[B51] He Y. Wang R. Dai X. Zhao Y. (2017a). On improving CRISPR for editing plant genes: ribozyme-mediated guide RNA production and fluorescence-based technology for isolating transgene-free mutants generated by CRISPR. Prog. Mol. Biol. Transl. Sci. 149, 151–166. doi: 10.1016/bs.pmbts.2017.03.012 28712495

[B52] He Y. Zhang T. Yang N. Xu M. Yan L. Wang L.. (2017b). Self-cleaving ribozymes enable the production of guide RNAs from unlimited choices of promoters for CRISPR/Cas9 mediated genome editing. J. Genet. Genomics 44, 469. doi: 10.1016/j.jgg.2017.08.003 28958488 PMC5736383

[B53] Hsu P. D. Scott D. A. Weinstein J. A. Ran F. A. Konermann S. Agarwala V.. (2013). DNA Targeting specificity of RNA-guided Cas9 nucleases. Nat. Biotechnol. 31, 827–832. doi: 10.1038/nbt.2647 23873081 PMC3969858

[B54] Huang X. Hilscher J. Stoger E. Christou P. Zhu C. (2021). Modification of cereal plant architecture by genome editing to improve yields. Plant Cell Rep. 40, 953–978. doi: 10.1007/s00299-021-02668-7 33559722

[B55] Huang Y. Shang M. Liu T. Wang K. (2022). High-throughput methods for genome editing: the more the better. Plant Physiol 188 (4), 1731–1745. doi: 10.1093/plphys/kiac017 35134245 PMC8968257

[B56] Ignacimuthu S. Arockiasamy S. Terada R. (2000). Genetic transformation of rice: current status and future prospects. Curr. Sci. 79 (2), 186–195.

[B57] Ithnin M. Vu W. T. Shin M.-G. Suryawanshi V. Sherbina K. Zolkafli S. H.. (2021). Genomic diversity and genome-wide association analysis related to yield and fatty acid composition of wild American oil palm. Plant Sci. 304, 110731. doi: 10.1016/j.plantsci.2020.110731 33568284

[B58] Izawati A. M. D. Parveez G. K. A. Masani M. Y. A. (2012). “Transformation of oil palm using agrobacterium tumefaciens,” in Transgenic plants (Humana Press), 177–188. 10.1007/978-1-61779-558-9_15 22351008

[B59] Jacobs T. B. Zhang N. Patel D. Martin G. B. (2017). Generation of a collection of mutant tomato lines using pooled CRISPR libraries. Plant Physiol. 174, 2023–2037. doi: 10.1104/pp.17.00489 28646085 PMC5543939

[B60] Jain M. (2015). Function genomics of abiotic stress tolerance in plants: A CRISPR approach. Front. Plant Sci. 6, 375. doi: 10.3389/fpls.2015.00375 26074938 PMC4445320

[B61] Jiang W. Zhou H. Bi H. Fromm M. Yang B. Weeks D. P. (2013). Demonstration of CRISPR/Cas9/sgRNA-mediated targeted gene modification in arabidopsis, tobacco, sorghum and rice. Nucleic Acids Res. 41, e188–e188. doi: 10.1093/nar/gkt780 23999092 PMC3814374

[B62] Jia H. Wang N. (2014). Xcc-facilitated agroinfiltration of citrus leaves: A tool for rapid functional analysis of transgenes in citrus leaves. Plant Cell Rep. 33, 1993–2001. doi: 10.1007/s00299-014-1673-9 25146436

[B63] Jia H. Xu J. Orbović V. Zhang Y. Wang N. (2017). Editing citrus genome *via* SaCas9/sgRNA system. Front. Plant Sci. 8, 2135. doi: 10.3389/fpls.2017.02135 29312390 PMC5732962

[B64] Jones A. Davies H. M. Voelker T. A. (1995). Palmitoyl-acyl carrier protein (ACP) thioesterase and the evolutionary origin of plant acyl-ACP thioesterases. Plant Cell 7, 359–371. doi: 10.1105/tpc.7.3.359 7734968 PMC160788

[B65] Jubrael J. M. Udupa S. M. Baum M. (2005). Assessment of AFLP-based genetic relationships among date palm (Phoenix dactylifera l.) varieties of Iraq. J. Am. Soc Hortic. Sci. 130, 442–447. doi: 10.21273/JASHS.130.3.442

[B66] Kadir A. P. G. (2003). Novel products from transgenic oil palm. AgBiotechNet 113, 1–9. doi: 10.1079/cabireviews20033177379

[B67] Kanchiswamy C. N. (2016). DNA-Free genome editing methods for targeted crop improvement. Plant Cell Rep. 35, 1469–1474. doi: 10.1007/s00299-016-1982-2 27100964

[B68] Kaur N. Alok A. Kaur N. Pandey P. Awasthi P. Tiwari S. (2018). CRISPR/Cas9-mediated efficient editing in phytoene desaturase (PDS) demonstrates precise manipulation in banana cv. Rasthali genome. Funct. Integrat. Genomics 18, 89–99. doi: 10.1007/s10142-017-0577-5 29188477

[B69] Khan F. S. Gan Z.-M. Li E.-Q. Ren M.-K. Hu C.-G. Zhang J.-Z. (2022). Transcriptomic and physiological analysis reveals interplay between salicylic acid and drought stress in citrus tree floral initiation. Planta 255, 1–22. doi: 10.1007/s00425-021-03801-2 34928452

[B70] Khatodia S. Bhatotia K. Passricha N. Khurana S. Tuteja N. (2016). The CRISPR/Cas genome-editing tool: Application in improvement of crops. Front. Plant Sci. 7, 506. doi: 10.3389/fpls.2016.00506 27148329 PMC4835450

[B71] Kim H. U. Jung S.-J. Lee K.-R. Kim E. H. Lee S.-M. Roh K. H.. (2014). Ectopic overexpression of castor bean LEAFY COTYLEDON2 (LEC2) in arabidopsis triggers the expression of genes that encode regulators of seed maturation and oil body proteins in vegetative tissues. FEBS Open Bio 4, 25–32. doi: 10.1016/j.fob.2013.11.003 PMC3863707 24363987

[B72] Kim H. Kim S.-T. Ryu J. Kang B.-C. Kim J.-S. Kim S.-G. (2017). CRISPR/Cpf1-mediated DNA-free plant genome editing. Nat. Commun. 8, 1–7. doi: 10.1038/ncomms14406 28205546 PMC5316869

[B73] Kumar A. Sharma A. C Upadhyaya K. (2016). Vegetable oil: nutritional and industrial perspective. Curr. Genom. 17, 230–240. doi: 10.2174/1389202917666160202220107 PMC4869010 27252590

[B74] Lantican D. V. Strickler S. R. Canama A. O. Gardoce R. R. Mueller L. A. Galvez H. F. (2019). De novo genome sequence assembly of dwarf coconut (Cocos nucifera L.’Catigan green dwarf’) provides insights into genomic variation between coconut types and related palm species. Genes Genom Genet. 9, 2377–2393. doi: 10.1534/g3.119.400215 PMC6686914 31167834

[B75] Lee R. T. H. Ng A. S. M. Ingham P. W. (2016). Ribozyme mediated gRNA generation for *in vitro* and *in vivo* CRISPR/Cas9 mutagenesis. PloS One 11, e0166020. doi: 10.1371/journal.pone.0166020 27832146 PMC5104441

[B76] Lei J. Dai P. Li J. Yang M. Li X. Zhang W.. (2021). Tissue-specific CRISPR/Cas9 system of cotton pollen with GhPLIMP2b and GhMYB24 promoters. J. Plant Biol. 64, 13–21. doi: 10.1007/s12374-020-09272-4

[B77] Liang Z. Chen K. Li T. Zhang Y. Wang Y. Zhao Q.. (2017). Efficient DNA-free genome editing of bread wheat using CRISPR/Cas9 ribonucleoprotein complexes. Nat. Commun. 8, 1–5. doi: 10.1038/ncomms14261 28098143 PMC5253684

[B78] Lian J. Schultz C. Cao M. Hamedirad M. Zhao H. (2019). Multi-functional genome-wide CRISPR system for high throughput genotype–phenotype mapping. Nat. Commun. 10, 1–10. doi: 10.1038/s41467-019-13621-4 31857575 PMC6923430

[B79] Li X. Jiang D. H. Yong K. Zhang D. B. (2007). Varied transcriptional efficiencies of multiple arabidopsis U6 small nuclear RNA genes. J. Integr. Plant Biol. 49, 222–229. doi: 10.1111/j.1744-7909.2007.00393.x

[B80] Li H. Li X. Xu Y. Liu H. He M. Tian X.. (2020). High-efficiency reduction of rice amylose content *via* CRISPR/Cas9-mediated base editing. Rice Sci. 27, 445. doi: 10.1016/j.rsci.2020.09.001

[B81] Limera C. Sabbadini S. Sweet J. B. Mezzetti B. (2017). New biotechnological tools for the genetic improvement of major woody fruit species. Front. Plant Sci. 8, 1418. doi: 10.3389/fpls.2017.01418 28861099 PMC5559511

[B82] Lineesha K. Antony G. (2021). “Genome editing: Prospects and challenges,” In The Coconut Genome (Springer), 191–203.

[B83] Li J.-F. Norville J. E. Aach J. Mccormack M. Zhang D. Bush J.. (2013). Multiplex and homologous recombination–mediated genome editing in arabidopsis and nicotiana benthamiana using guide RNA and Cas9. Nat. Biotechnol. 31, 688–691. doi: 10.1038/nbt.2654 23929339 PMC4078740

[B84] Liu Z. Dong H. Cui Y. Cong L. Zhang D. (2020). Application of different types of CRISPR/Cas-based systems in bacteria. Microb. Cell Factories 19, 1–14. doi: 10.1186/s12934-020-01431-z PMC7470686 32883277

[B85] Liu X. Homma A. Sayadi J. Yang S. Ohashi J. Takumi T. (2016). Sequence features associated with the cleavage efficiency of CRISPR/Cas9 system. Sci. Rep. 6, 1–9. doi: 10.1038/srep19675 26813419 PMC4728555

[B86] Li J.-W. Zeng T. Xu Z.-Z. Li J.-J. Hu H. Yu Q.. (2022). Ribozyme-mediated CRISPR/Cas9 gene editing in pyrethrum (Tanacetum cinerariifolium) hairy roots using a RNA polymerase II-dependent promoter. Plant Methods 18, 32. doi: 10.1186/s13007-022-00863-5 35292048 PMC8925089

[B87] Li J.-F. Zhang D. Sheen J. (2014). “Cas9-based genome editing in arabidopsis and tobacco,” In Methods in enzymology (Academic Press) 546, 459–472. 25398353 10.1016/B978-0-12-801185-0.00022-2

[B88] Long L. Guo D.-D. Gao W. Yang W.-W. Hou L.-P. Ma X.-N.. (2018). Optimization of CRISPR/Cas9 genome editing in cotton by improved sgRNA expression. Plant Methods 14, 1–9. doi: 10.1186/s13007-018-0353-0 30305839 PMC6169012

[B89] Lu H. P. Liu S. M. Xu S. L. Chen W. Y. Zhou X. Tan Y. Y.. (2017). CRISPR-s: an active interference element for a rapid and inexpensive selection of genome-edited, transgene-free rice plants. Plant Biotechnol. J. 15, 1371. doi: 10.1111/pbi.12788 28688132 PMC5633759

[B90] Madon M. Clyde M. Hashim H. Mohd Yusuf Y. Mat H. Saratha S. (2005). Polyploidy induction of oil palm through colchicine and oryzalin treatments. J. Oil Palm Res. 17, 110.

[B91] Malnoy M. Viola R. Jung M.-H. Koo O.-J. Kim S. Kim J.-S.. (2016). DNA-Free genetically edited grapevine and apple protoplast using CRISPR/Cas9 ribonucleoproteins. Front. Plant Sci. 7, 1904. doi: 10.3389/fpls.2016.01904 28066464 PMC5170842

[B92] Manickavasagan A. Essa M. M. Sukumar E. Eds. (2012). Dates: production, processing, food, and medicinal values (CRC Press).

[B93] Mao Y. Zhang H. Xu N. Zhang B. Gou F. Zhu J.-K. (2013). Application of the CRISPR–cas system for efficient genome engineering in plants. Mol. Plant 6, 2008–2011. doi: 10.1093/mp/sst121 23963532 PMC3916745

[B94] Marz M. Stadler P. F. (2009). Comparative analysis of eukaryotic U3 snoRNA. RNA Biol. 6, 503–507. doi: 10.4161/rna.6.5.9607 19875933

[B95] Masani M. Y. A. Noll G. Parveez G. K. A. Sambanthamurthi R. Prüfer D. (2013). Regeneration of viable oil palm plants from protoplasts by optimizing media components, growth regulators and cultivation procedures. Plant Sci. 210, 118–127. doi: 10.1016/j.plantsci.2013.05.021 23849119

[B96] Masani M. Y. A. Noll G. A. Parveez G. K. A. Sambanthamurthi R. Prüfer D. (2014). Efficient transformation of oil palm protoplasts by PEG-mediated transfection and DNA microinjection. PloS One 9, e96831. doi: 10.1371/journal.pone.0096831 24821306 PMC4018445

[B97] Masani M. Y. A. Parveez G. K. A. Noll G. Fizree M. D. Sambanthamurthi R. Pruefer D. (2022). Protoplast isolation and transformation in oil palm. In: Protoplast Technology. (New York, NY: Humana) pp. 187–202. 10.1007/978-1-0716-2164-6_14 35258834

[B98] Masli D. I. A. Kadir A. P. G. Yunus A. M. M. (2009). Transformation of oil palm using agrobacterium tumefaciens. J. Oil Palm Res. 21, 643–652.

[B99] Mathew L. S. Seidel M. A. George B. Mathew S. Spannagl M. Haberer G.. (2015). A genome-wide survey of date palm cultivars supports two major subpopulations in phoenix dactylifera. G3: Genes Genom Genet. 5, 1429–1438. doi: 10.1534/g3.115.018341 PMC4502377 25957276

[B100] Ma X. Zhang Q. Zhu Q. Liu W. Chen Y. Qiu R.. (2015). A robust CRISPR/Cas9 system for convenient, high-efficiency multiplex genome editing in monocot and dicot plants. Mol. Plant 8, 1274–1284. doi: 10.1016/j.molp.2015.04.007 25917172

[B101] Meerow A. W. Krueger R. R. Singh R. Low E.-T. L. Ithnin M. Ooi L. C.-L. (2012). “Coconut, date, and oil palm genomics,” in Genomics of tree crops (New York, NY: Springer), 299–351.

[B102] Meng X. Yu H. Zhang Y. Zhuang F. Song X. Gao S.. (2017). Construction of a genome-wide mutant library in rice using CRISPR/Cas9. Mol. Plant 10, 1238–1241. doi: 10.1016/j.molp.2017.06.006 28645639

[B103] Metje-Sprink J. Menz J. Modrzejewski D. Sprink T. (2019). DNA-Free genome editing: past, present and future. Front. Plant Sci. 9, 1957. doi: 10.3389/fpls.2018.01957 30693009 PMC6339908

[B104] Molla K. A. Yang Y. (2019). CRISPR/Cas-mediated base editing: technical considerations and practical applications. Trends Biotechnol. 37, 1121–1142. doi: 10.1016/j.tibtech.2019.03.008 30995964

[B105] Moradpour M. Abdulah S. N. A. (2020). CRISPR/dC as9 platforms in plants: strategies and applications beyond genome editing. Plant Biotechol J. 18, 32–44. doi: 10.1111/pbi.13232 PMC6920162 31392820

[B106] Mousavi M. Mousavi A. Habashi A. Arzani K. Dehsara B. (2014a). Transient transformation of date palm *via* agrobacterium-mediated and particle bombardment. Emir J. Food Agric. 26, 528–538. doi: 10.9755/ejfa.v26i5.15722

[B107] Mousavi M. Mousavi A. Habashi A. A. Arzani K. Dehsara B. Brajeh M. (2017). “Genetic transformation of date palm via microprojectile bombardment,” in Date palm biotechnology protocols volume I (New York, NY: Humana Press), 269–280. 10.1007/978-1-4939-7156-5_22 28755352

[B108] Mousavi M. Mousavi A. Habashi A. A. Dehsara B. (2014b). Genetic transformation of date palm (Phoenix dactylifera l. cv.’Estamaran’) *via* particle bombardment. Mol. Biol. Rep. 41, 8185–8194. doi: 10.1007/s11033-014-3720-6 25200434

[B109] Nakajima I. Ban Y. Azuma A. Onoue N. Moriguchi T. Yamamoto T.. (2017). CRISPR/Cas9-mediated targeted mutagenesis in grape. PloS One 12, e0177966. doi: 10.1371/journal.pone.0177966 28542349 PMC5436839

[B110] Nekrasov V. Staskawicz B. Weigel D. Jones J. D. Kamoun S. (2013). Targeted mutagenesis in the model plant nicotiana benthamiana using Cas9 RNA-guided endonuclease. Nat. Biotechnol. 31, 691–693. doi: 10.1038/nbt.2655 23929340

[B111] Nguyen Q. T. Bandupriya H. López-Villalobos A. Sisunandar S. Foale M. Adkins S. W. (2015). Tissue culture and associated biotechnological interventions for the improvement of coconut (Cocos nucifera l.): A review. Planta 242, 1059–1076. 26189000 10.1007/s00425-015-2362-9

[B112] Nishitani C. Hirai N. Komori S. Wada M. Okada K. Osakabe K.. (2016). Efficient genome editing in apple using a CRISPR/Cas9 system. Sci. Rep. 6, 1–8. doi: 10.1038/srep31481 27530958 PMC4987624

[B113] Parveez G. K. A. Christou P. (1998). Biolistic-mediated DNA delivery and isolation of transgenic oil palm (Elaeis guineensis jacq.) embryogenic callus cultures. J. Oil Palm Res. 10, 29–38.

[B114] Parveez G. K. A. Masri M. M. Zainal A. Majid N. I. A. Yunus A. M. M. Fadilah H. H.. (2000). Transgenic oil palm: production and projection (Portland Press Ltd). 11171275

[B115] Pattanayak V. Lin S. Guilinger J. P. Ma E. Doudna J. A. Liu D. R. (2013). High-throughput profiling of off-target DNA cleavage reveals RNA-programmed Cas9 nuclease specificity. Nat. Biotechnol. 31, 839–843. doi: 10.1038/nbt.2673 23934178 PMC3782611

[B116] Perera S. (2014). “Oil palm and coconut,” in Alien gene transfer in crop plants, vol. 2. (New York NY: Springer), 231–252.

[B117] Peterson B. A. Haak D. C. Nishimura M. T. Teixeira P. J. James S. R. Dangl J. L.. (2016). Genome-wide assessment of efficiency and specificity in CRISPR/Cas9 mediated multiple site targeting in arabidopsis. PloS One 11, e0162169. doi: 10.1371/journal.pone.0162169 27622539 PMC5021288

[B118] Rajesh M. Ramesh S. Perera L. Kole C. Eds. (2021). The coconut genome (Springer International Publishing).

[B119] Rekik I. Chaabene Z. Grubb C. D. Drira N. Cheour F. Elleuch A. (2015). In silico characterization and molecular modeling of double-strand break repair protein MRE11 from phoenix dactylifera v deglet nour. Theor. Biol. Med. Model. 12, 1–14. doi: 10.1186/s12976-015-0013-2 26541955 PMC4635681

[B120] Ren C. Liu Y. Guo Y. Duan W. Fan P. Li S.. (2021). Optimizing the CRISPR/Cas9 system for genome editing in grape by using grape promoters. Hortic. Res. 8, 1–12. doi: 10.1038/s41438-021-00489-z 33642575 PMC7917103

[B121] Reynolds K. B. Cullerne D. P. El Tahchy A. Rolland V. Blanchard C. L. Wood C. C.. (2019). Identification of genes involved in lipid biosynthesis through *de novo* transcriptome assembly from Cocos nucifera developing endosperm. Plant Cell Physiol. 60, 945–960. doi: 10.1093/pcp/pcy247 30608545 PMC6498750

[B122] Reynolds K. B. Taylor M. C. Zhou X.-R. Vanhercke T. Wood C. C. Blanchard C. L.. (2015). Metabolic engineering of medium-chain fatty acid biosynthesis in nicotiana benthamiana plant leaf lipids. Front. Plant Sci. 6, 164. doi: 10.3389/fpls.2015.00164 25852716 PMC4371700

[B123] Ritchie H. Roser M. (2020). “Agricultural production,” in Our world in data.

[B124] Ritchie H. Roser M. (2021). “Forests and deforestation,” in Our world in data. Available at: https://ourworldindata.org/.

[B125] Roeder R. G. Rutter W. J. (1969). Multiple forms of DNA-dependent RNA polymerase in eukaryotic organisms. Nature 224, 234–237. doi: 10.1038/224234a0 5344598

[B126] Rohde W. Sniady V. Herran A. Estioko L. Sinje S. Marseillac N.. (2002). Construction and exploitation of high density DNA marker and physical maps in the perennial tropical oil crops coconut and oil palm: From biotechnology towards marker-assisted breeding. Burotrop Bull. 20, 13–14.

[B127] Sabir J. S. Arasappan D. Bahieldin A. Abo-Aba S. Bafeel S. Zari T. A.. (2014). Whole mitochondrial and plastid genome SNP analysis of nine date palm cultivars reveals plastid heteroplasmy and close phylogenetic relationships among cultivars. PloS One 9, e94158. doi: 10.1371/journal.pone.0094158 24718264 PMC3981771

[B128] Safari F. Zare K. Negahdaripour M. Barekati-Mowahed M. Ghasemi Y. (2019). CRISPR Cpf1 proteins: structure, function and implications for genome editing. Cell Biosci. 9, 1–21. doi: 10.1186/s13578-019-0298-7 31086658 PMC6507119

[B129] Saker M. Kumlehn J. Ghareeb H. (2009). Factors influencing transient expression of agrobacterium-mediated transformation of GUS gene in embryogenic callus of date palm. Adv. Hortic. Sci. 23, 150–157.

[B130] Salomón-Torres R. Valdez-Salas B. Norzagaray-Plasencia S. (2021). “Date palm: Source of foods, sweets and beverages,” in The date palm genome, vol. 2. (Cham: Springer), 3–26.

[B131] Salsman J. Dellaire G. (2017). Precision genome editing in the CRISPR era. Biochem. Cell Biol. 95, 187–201. doi: 10.1139/bcb-2016-0137 28177771

[B132] Sambanthamurthi R. Abdullah S. Kadir A. (2002). Genetic manipulation of the oil palm-challenges and prospects. Planter 78, 547–564.

[B133] Sattar M. N. Iqbal Z. Al-Khayri J. M. (2021). “CRISPR-cas based precision breeding in date palm: Future applications,” in The date palm genome, vol. 2. (Cham: Springer), 169–199.

[B134] Sattar M. N. Iqbal Z. Tahir M. N. Shahid M. S. Khurshid M. Al-Khateeb A. A.. (2017). CRISPR/Cas9: A practical approach in date palm genome editing. Front. Plant Sci. 8, 1469. doi: 10.3389/fpls.2017.01469 28878801 PMC5572371

[B135] Sattler M. C. Carvalho C. R. Clarindo W. R. (2016). The polyploidy and its key role in plant breeding. Planta 243, 281–296. doi: 10.1007/s00425-015-2450-x 26715561

[B136] Sayer J. Ghazoul J. Nelson P. Boedhihartono A. K. (2012). Oil palm expansion transforms tropical landscapes and livelihoods. Glob. Food Sec. 1, 114–119. doi: 10.1016/j.gfs.2012.10.003

[B137] Serganov A. Patel D. J. (2007). Ribozymes, riboswitches and beyond: regulation of gene expression without proteins. Nat. Rev. Genet. 8, 776–790. doi: 10.1038/nrg2172 17846637 PMC4689321

[B138] Shafique Khan F. Zeng R.-F. Gan Z.-M. Zhang J.-Z. Hu C.-G. (2021). Genome-wide identification and expression profiling of the WOX gene family in citrus sinensis and functional analysis of a CsWUS member. Int. J. Mol. Sci. 22, 4919. doi: 10.3390/ijms22094919 34066408 PMC8124563

[B139] Shan Q. Wang Y. Li J. Zhang Y. Chen K. Liang Z.. (2013). Targeted genome modification of crop plants using a CRISPR-cas system. Nat. Biotechnol. 31, 686–688. doi: 10.1038/nbt.2650 23929338

[B140] Singha D. L. Das D. Sarki Y. N. Chowdhury N. Sharma M. Maharana J.. (2022). Harnessing tissue-specific genome editing in plants through CRISPR/Cas system: Current state and future prospects. Planta 255, 1–17. doi: 10.1007/s00425-021-03811-0 34962611

[B141] Singh R. Lan C. P. Ithnin M. Ramli U. S. (2018). “Advances in marker-assisted breeding of oil palm Malaysian palm oil board, Malaysia,” in Achieving sustainable cultivation of oil palm, vol. 1. (Burleigh Dodds Science Publishing), 161–184.

[B142] Sprink T. Eriksson D. Schiemann J. Hartung F. (2016). Regulatory hurdles for genome editing: process-vs. product-based approaches in different regulatory contexts. Plant Cell Rep. 35, 1493–1506. doi: 10.1007/s00299-016-1990-2 27142995 PMC4903111

[B143] Stoutjesdijk P. Hurlestone C. Singh S. Green A. (2000). High-oleic acid Australian brassica napus and b. juncea varieties produced by co-suppression of endogenous Δ12-desaturases (Portland Press). 11171263

[B144] Stracker T. H. Petrini J. H. (2011). The MRE11 complex: starting from the ends. Nat. Rev. Mol. Cell Biol. 12, 90–103. doi: 10.1038/nrm3047 21252998 PMC3905242

[B145] Subhi S. M. Tahir N. I. Abd Rasid O. Ramli U. S. Othman A. Masani M. Y. A.. (2017). Post-genomic technologies for the advancement of oil palm research. J. Oil Palm Res. 29, 469–486. doi: 10.21894/jopr.2017.00013

[B146] Sun R. Gao L. Mi Z. Zheng Y. Li D. (2020). CnMADS1, a MADS transcription factor, positively modulates cell proliferation and lipid metabolism in the endosperm of coconut (Cocos nucifera l.). Planta 252, 1–13. doi: 10.1007/s00425-020-03490-3 33040224

[B147] Tang X. Ren Q. Yang L. Bao Y. Zhong Z. He Y.. (2019). Single transcript unit CRISPR 2.0 systems for robust Cas9 and Cas12a mediated plant genome editing. Plant Biotechnol. J. 17, 1431–1445. doi: 10.1111/pbi.13068 30582653 PMC6576101

[B148] Te-chato S. S. S. (2012). Ploidy induction through secondary somatic embryo (SSE) of oil palm by colchicine treatment. J. Agri Technol. 8, 337–352.

[B149] Teixeira J. Söndahl M. Nakamura T. Kirby E. (1995). Establishment of oil palm cell suspensions and plant regeneration. Plant Cell Tissue Organ Cult. 40, 105–111. doi: 10.1007/BF00037662

[B150] Ting N.-C. Sherbina K. Khoo J.-S. Kamaruddin K. Chan P.-L. Chan K.-L.. (2020). Expression of fatty acid and triacylglycerol synthesis genes in interspecific hybrids of oil palm. Sci. Rep. 10, 1–15. doi: 10.1038/s41598-020-73170-5 33004875 PMC7529811

[B151] Triozzi P. M. Schmidt H. W. Dervinis C. Kirst M. Conde D. (2021). Simple, efficient and open-source CRISPR/Cas9 strategy for multi-site genome editing in populus tremula× alba. Tree Physiol. 41, 2216–2227. doi: 10.1093/treephys/tpab066 33960379 PMC8597961

[B152] Tsai C.-J. Xue L.-J. (2015). CRISPRing into the woods. GM Crops Food 6, 206–215. doi: 10.1080/21645698.2015.1091553 26357840 PMC5033219

[B153] Uniyal A. P. Mansotra K. Yadav S. K. Kumar V. (2019). An overview of designing and selection of sgRNAs for precise genome editing by the CRISPR-Cas9 system in plants. 3 Biotech. 9, 1–19. doi: 10.1007/s13205-019-1760-2 PMC6529479 31139538

[B154] Vanhercke T. El Tahchy A. Shrestha P. Zhou X.-R. Singh S. P. Petrie J. R. (2013). Synergistic effect of WRI1 and DGAT1 coexpression on triacylglycerol biosynthesis in plants. FEBS Lett. 587, 364–369. doi: 10.1016/j.febslet.2012.12.018 23313251

[B155] Venema J. Vos H. R. Faber A. W. Van Venrooij W. J. Raué H. A. (2000). Yeast Rrp9p is an evolutionarily conserved U3 snoRNP protein essential for early pre-rRNA processing cleavages and requires box c for its association. Rna 6, 1660–1671. doi: 10.1017/S1355838200001369 11105764 PMC1370034

[B156] Wahid M. B. Abdullah S. N. A. Ie H. (2005). Oil palm–achievements and potential. Plant Prod. Sci. 8, 288–297. doi: 10.1626/pps.8.288

[B157] Walton R. T. Christie K. A. Whittaker M. N. Kleinstiver B. P. (2020). Unconstrained genome targeting with near-PAMless engineered CRISPR-Cas9 variants. Science 368, 290–296. doi: 10.1126/science.aba8853 32217751 PMC7297043

[B158] Wang Y. Ihase L. O. Htwe Y. M. Shi P. Zhang D. Li D.. (2020). Development of sex-linked SSR marker in the genus phoenix and validation in p. dactylifera. Crop Sci. 60, 2452–2466. doi: 10.1002/csc2.20187

[B159] Wang Z. Wang S. Li D. Zhang Q. Li L. Zhong C.. (2018). Optimized paired-sgRNA/Cas9 cloning and expression cassette triggers high-efficiency multiplex genome editing in kiwifruit. Plant Biotechnol. J. 16, 1424–1433. doi: 10.1111/pbi.12884 29331077 PMC6041439

[B160] Weckx S. Inzé D. Maene L. (2019). Tissue culture of oil palm: Finding the balance between mass propagation and somaclonal variation. Front. Plant Sci. 10, 722. doi: 10.3389/fpls.2019.00722 31214232 PMC6558080

[B161] Wolter F. Puchta H. (2018). The CRISPR/Cas revolution reaches the RNA world: Cas13, a new Swiss army knife for plant biologists. Plant J. 94, 767–775. doi: 10.1111/tpj.13899 29575326

[B162] Woo J. W. Kim J. Kwon S. I. Corvalán C. Cho S. W. Kim H.. (2015). DNA-Free genome editing in plants with preassembled CRISPR-Cas9 ribonucleoproteins. Nat. Biotechnol. 33, 1162–1164. doi: 10.1038/nbt.3389 26479191

[B163] Xia W. Luo T. Dou Y. Zhang W. Mason A. S. Huang D.. (2019). Identification and validation of candidate genes involved in fatty acid content in oil palm by genome-wide association analysis. Front. Plant Sci. 10, 1263. doi: 10.3389/fpls.2019.01263 31681369 PMC6804545

[B164] Xiao Y. Xu P. Fan H. Baudouin L. Xia W. Bocs S.. (2017). The genome draft of coconut (Cocos nucifera). Gigascience 6, gix095. doi: 10.1093/gigascience/gix095 PMC5714197 29048487

[B165] Xie K. Yang Y. (2013). RNA-Guided genome editing in plants using a CRISPR–cas system. Mol. Plant 6, 1975–1983. doi: 10.1093/mp/sst119 23956122

[B166] Xu X. Liu X. Ge S. Jensen J. D. Hu F. Li X.. (2012). Resequencing 50 accessions of cultivated and wild rice yields markers for identifying agronomically important genes. Nat. Biotechnol. 30, 105–111. doi: 10.1038/nbt.2050 22158310

[B167] Yang M. Zhang X. Liu G. Yin Y. Chen K. Yun Q.. (2010). The complete chloroplast genome sequence of date palm (Phoenix dactylifera l.). PloS One 5, e12762. doi: 10.1371/journal.pone.0012762 20856810 PMC2939885

[B168] Yarra R. Cao H. Jin L. Mengdi Y. Zhou L. (2020). CRISPR/Cas mediated base editing: a practical approach for genome editing in oil palm. Biotech 10, 1–7. doi: 10.1007/s13205-020-02302-5 32566443 PMC7295873

[B169] Yeap W. C. Lee F. C. Shabari Shan D. K. Musa H. Appleton D. R. Kulaveerasingam H. (2017). WRI 1-1, ABI 5, NF-YA 3 and NF-YC 2 increase oil biosynthesis in coordination with hormonal signaling during fruit development in oil palm. Plant J. 91, 97–113. doi: 10.1111/tpj.13549 28370622

[B170] Yeap W.-C. Norkhairunnisa K. C. M. Norfadzilah J. Muad M. R. Appleton D. R. Harikrishna K. (2021). An efficient clustered regularly interspaced short palindromic repeat (CRISPR)/CRISPR-associated protein 9 mutagenesis system for oil palm (Elaeis guineensis). Front. Plant Sci. 12, 773656–773656. doi: 10.3389/fpls.2021.773656 34880893 PMC8647858

[B171] Yue J.-J. Hong C.-Y. Wei P. Tsai Y.-C. Lin C.-S. (2020). How to start your monocot CRISPR/Cas project: plasmid design, efficiency detection, and offspring analysis. Rice 13, 1–13. doi: 10.1186/s12284-019-0354-2 32016561 PMC6997315

[B172] Yue G. H. Ye B. Q. Lee M. (2021). Molecular approaches for improving oil palm for oil. Mol. Breed. 41, 1–17. doi: 10.1007/s11032-021-01218-z PMC10236033 37309424

[B173] Zafar S. A. Zaidi S.S.-E.-A. Gaba Y. Singla-Pareek S. L. Dhankher O. P. Li X.. (2020). Engineering abiotic stress tolerance *via* CRISPR/Cas-mediated genome editing. J. Exp. Bot. 71, 470–479. doi: 10.1093/jxb/erz476 31644801

[B174] Zetsche B. Gootenberg J. S. Abudayyeh O. O. Slaymaker I. M. Makarova K. S. Essletzbichler P.. (2015). Cpf1 is a single RNA-guided endonuclease of a class 2 CRISPR-cas system. Cell 163, 759–771. doi: 10.1016/j.cell.2015.09.038 26422227 PMC4638220

[B175] Zhang Y. Bai B. Lee M. Alfiko Y. Suwanto A. Yue G. H. (2018). Cloning and characterization of EgGDSL, a gene associated with oil content in oil palm. Sci. Rep. 8, 1–11. doi: 10.1038/s41598-018-29492-6 30061629 PMC6065316

[B176] Zhang F. Leblanc C. Irish V. F. Jacob Y. (2017b). Rapid and efficient CRISPR/Cas9 gene editing in citrus using the YAO promoter. Plant Cell Rep. 36, 1883–1887. doi: 10.1007/s00299-017-2202-4 28864834

[B177] Zhang Y. Liang Z. Zong Y. Wang Y. Liu J. Chen K.. (2016). Efficient and transgene-free genome editing in wheat through transient expression of CRISPR/Cas9 DNA or RNA. Nat. Commun. 7, 1–8. doi: 10.1038/ncomms12617 PMC5007326 27558837

[B178] Zhang J. Liu H. Sun J. Li B. Zhu Q. Chen S.. (2012). Arabidopsis fatty acid desaturase FAD2 is required for salt tolerance during seed germination and early seedling growth. PloS One 7, e30355. doi: 10.1371/journal.pone.0030355 22279586 PMC3261201

[B179] Zhang D. Zhang H. Li T. Chen K. Qiu J.-L. Gao C. (2017a). Perfectly matched 20-nucleotide guide RNA sequences enable robust genome editing using high-fidelity SpCas9 nucleases. Genome Biol. 18, 1–7. doi: 10.1186/s13059-017-1325-9 29020979 PMC5637269

