# Supplementary material for: Is CRISPR/Cas9 a way forward to fast-track genetic improvement in commercial palms? Prospects and limits
Source: Front Plant Sci. 2022 Dec 12;13:1042828. doi: 10.3389/fpls.2022.1042828 (PMC9791139; doi:10.3389/fpls.2022.1042828)
Supplement: Supplementary file 1 [file Table_1.docx]

| **Tool name** | **Web address** | **Reference** |
| --- | --- | --- |
| CHOPCHOP | https://chopchop.rc.fas.harvard.edu/ | (Montague et al., 2014) |
| CCTop | http://crispr.cos.uni-heidelberg.de/ | (Stemmer et al., 2015) |
| CRISPR Design | http://crispr.mit.edu/ | (Hsu et al., 2013) |
| WU-CRISPR | http://crisprdb.org /wu-crispr/ | (Wong et al., 2015) |
| WGE CRISPR Finder | https://wge.stemcell.sanger.ac.uk/find_crisprs_id | (Hodgkins et al., 2015) |
| CRISPy CHO | staff.biosustain.dtu.dk/laeb/crispy | (Ronda et al., 2014) |
| sgRNAcas9 | http://www.biootools.com/col.jsp?id=14 | (Xie et al., 2014) |
| E-crisp | http://www.e-crisp.org/E-CRISP/ | (Heigwer et al., 2014) |
| CGAT | http://cbc.gdcb.iastate.edu/cgat/ | (Brazelton Jr et al., 2015) |
| Crispr-P | http://cbi.hzau.edu.cn/crispr/ | (Lei et al., 2014) |
| CrisprGE | http://crdd.osdd.net/servers/crisprge/ | (Kaur et al., 2015) |

**TABLE 1.** CRISPR sgRNA design tools
